# Tomato domestication rather than subsequent breeding events reduces microbial associations related to phosphorus recovery

**DOI:** 10.1038/s41598-024-60775-3

**Published:** 2024-04-30

**Authors:** Mary M. Dixon, Antisar Afkairin, Jessica G. Davis, Jessica Chitwood-Brown, Cassidy M. Buchanan, James A. Ippolito, Daniel K. Manter, Jorge M. Vivanco

**Affiliations:** 1 Department of Horticulture and Landscape Architecture, Colorado State University, Fort Collins, CO USA; 2 Department of Soil and Crop Sciences, Colorado State University, Fort Collins, CO USA; 3 United States Department of Agriculture–Agricultural Research Service, Soil Management and Sugar Beet Research, Fort Collins, CO USA; 4 School of Environment and Natural Resources, The Ohio State University, Columbus, OH USA

**Keywords:** Tomato, Phosphorus recovery, Domestication, Phosphorus solubilizing bacteria, Plant domestication, Abiotic

## Abstract

Legacy phosphorus (P) is a reservoir of sparingly available P, and its recovery could enhance sustainable use of nonrenewable mineral fertilizers. Domestication has affected P acquisition, but it is unknown if subsequent breeding efforts, like the Green Revolution (GR), had a similar effect. We examined how domestication and breeding events altered P acquisition by growing wild, traditional (pre-GR), and modern (post-GR) tomato in soil with legacy P but low bioavailable P. Wild tomatoes, particularly accession LA0716 (*Solanum pennellii*), heavily cultured rhizosphere P solubilizers, suggesting reliance on microbial associations to acquire P. Wild tomato also had a greater abundance of other putatively beneficial bacteria, including those that produce chelating agents and antibiotic compounds. Although wild tomatoes had a high abundance of these P solubilizers, they had lower relative biomass and greater P stress factor than traditional or modern tomato. Compared to wild tomato, domesticated tomato was more tolerant to P deficiency, and both cultivated groups had a similar rhizosphere bacterial community composition. Ultimately, this study suggests that while domestication changed tomato P recovery by reducing microbial associations, subsequent breeding processes have not further impacted microbial P acquisition mechanisms. Selecting microbial P-related traits that diminished with domestication may therefore increase legacy P solubilization.

## Introduction

Phosphorus (P), a plant-essential nutrient, undergoes soil processes, such as immobilization, precipitation, and adsorption, that reduce bioavailability^1^. Thus, to meet crop demand, modern cropping systems often rely on high applications of synthetic P fertilizers, which are sourced from nonrenewable rock phosphate^1,2^. Further, soil reactions that reduce bioavailability result in soil P accumulation in agroecosystems^2^. This growing pool of sparingly available legacy P provides a potential reservoir of nutrients which can offset inorganic P fertilization and reduce environmental risks associated with over fertilization, such as eutrophication^3^. Therefore, there is a need to improve P acquisition from legacy P pools.

Legacy P is sparingly available, but plant–microbe interactions promote P solubilization^4^. Exudation of root-derived secondary metabolites regulate these interactions, resulting in transformation of P from unavailable to bioavailable forms^5,6^. Root exudation may directly solubilize P or promote P-solubilizing microbial growth^7,8^. The ability of plants to form associations with P-solubilizing microbes varies between and within species^9–11^. Thus, strategies to enhance root-associated microbial interactions for the purpose of P solubilization are needed to reduce reliance on nonrenewable P fertilizers.

Root-associated microbiomes are impacted by numerous factors including exogenous P supply, soil properties, types of fertilization, plant development, and genotype^12–16^. For instance, tomato (*Solanum* spp.) rhizosphere bacterial communities vary between wild and cultivated groups^17,18^. Under fully fertilized conditions, groups of beneficial bacteria changed in abundance in the rhizosphere of successively planted tomato, in which wild tomato enriched *Rhizobium* and *Massilia* growth*,* and modern varieties enriched *Pseudomonas*^19^. Separation of the microbial communities may be due to changes in long-term plant breeding goals and agricultural practices which have, over time, impacted soil fertility and the rhizosphere microbiome^20–22^.

At domestication, desired traits were selected which were controlled by a limited number of alleles, resulting in a reduction in genetic diversity^23^. As breeding programs advanced, particularly during the Green Revolution (GR), cropping programs became reliant on increased use of inorganic fertilizers^24^. The Green Revolution resulted in high-yielding crops and intensive fertilization regimens which inadvertently affected rhizosphere community composition in crops such as wheat^22,25^. It is unknown if root-microbial associations related to nutrient solubilization are varied between pre-GR (traditional) and post-GR (modern) varieties of crops and how these abilities compare to wild crop relatives. Comparisons between wild, traditional, and modern varieties are therefore needed to discern differences in P acquisition.

To determine how two key plant breeding events (domestication and the GR) influenced P acquisition, representative wild, traditional, and modern tomato varieties were grown in sufficient and insufficient P conditions. Those varieties were grown in soil with low bioavailable P with the purpose of testing the ability of the plants to induce microbial associations related to P solubilization of legacy P. The overarching objective of this study was to examine soil bacteria and tomato domestication group interactions that relate to soil P solubilization and decomposition. It was hypothesized that tolerance to P deficiency and P solubilizing bacterial associations would differ among domestication groups. Ultimately, the varied performances of each domestication group in response to P deficiency elucidated how influential domestication and breeding are regarding rhizosphere P acquisition strategies.

## Results

### Divergent plant growth in P deficiency across a domestication gradient

**Figure 1:**
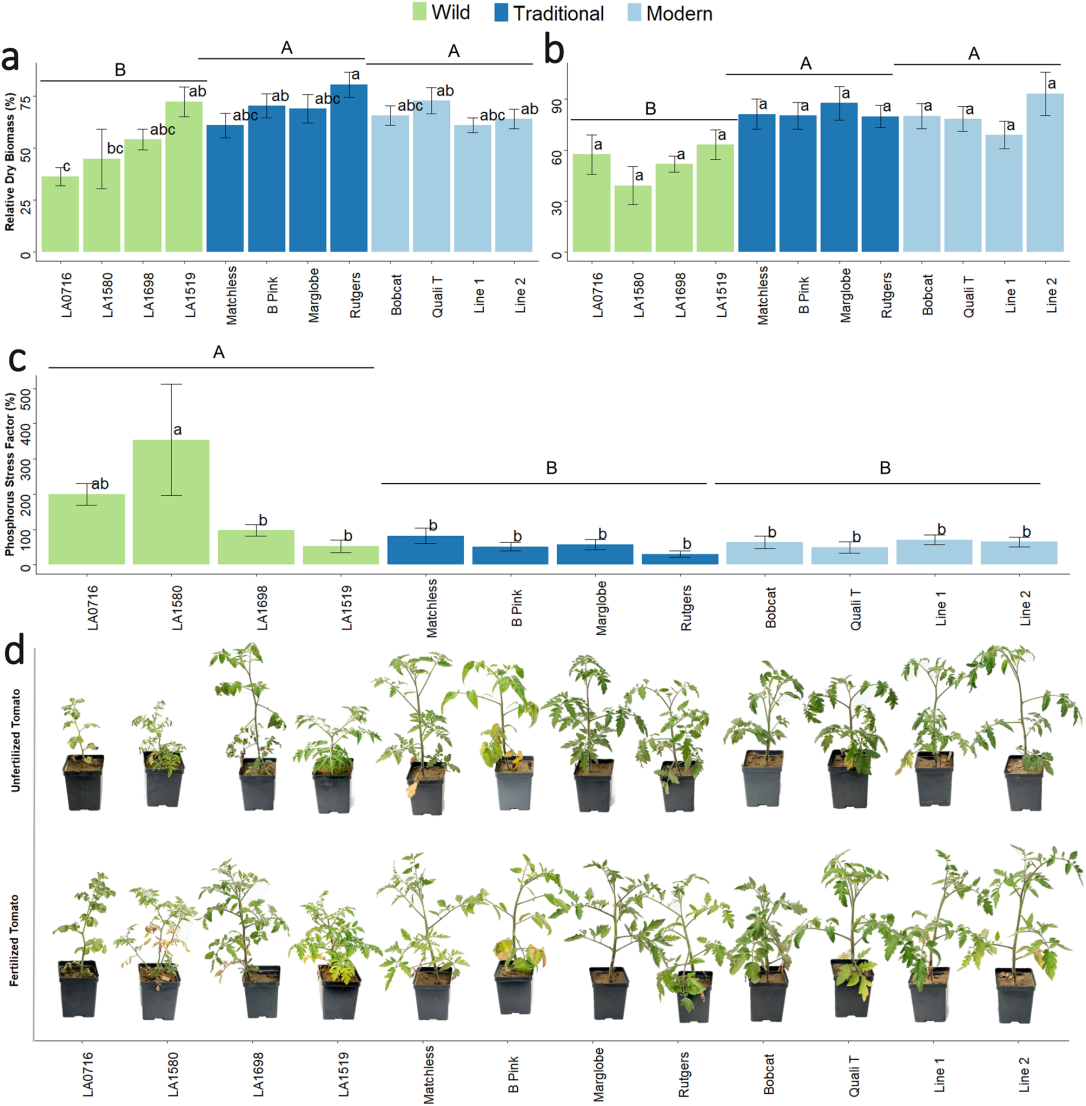
Phosphorus deficiency effects on tomato growth. (**a**) Relative dry shoot biomass. An ANOVA showed differences in for the Domestication main effect (df = 2, F = 7.107, p = 0.001) and for the Domestication-Variety interaction (df = 9, F = 2.385, p = 0.017). (**b**) Relative dry root biomass. An ANOVA showed differences in for the Domestication main effect (df = 2, F = 5.31, p = 0.006) and for the Domestication-Variety interaction (df = 9, F = 2.385, p = 0.017). (**c**) Phosphorus stress factor. An ANOVA showed differences in for the Domestication main effect (df = 2, F = 7.318, p = 0.001) and for the Domestication-Variety interaction (df = 9, F = 2.58, p = 0.010). (**d**) Representative samples of each accession. The top row shows tomatoes grown in the low phosphorus treatment, and the bottom row shows tomatoes grown in the sufficient phosphorus treatment. Different colored bars indicate the level of domestication: wild (green), traditional (dark blue), and modern (light blue). An analysis of variance (ANOVA) was performed with a Tukey HSD test for means comparison. Different lowercase letters represent significant differences (p < 0.05) in tomato genotype. Different uppercase letters represent significant differences (p < 0.05) in tomato domestication group. Treatments sharing a common letter are not significantly different. Presented as mean ± SEM.

Domestication (F = 7.11, p = 0.001) and variety (F = 2.39, p = 0.02) significantly changed relative shoot biomass (Fig. 1a). Traditional varieties, which were developed around the start of the 1900s (Supplemental Table 1), and modern varieties, which were developed after the 2000s (Supplemental Table 1), had a greater relative shoot biomass as compared to wild varieties (Fig. 1a). LA1519, ‘Brandywine Pink’ (henceforth “B Pink”), ‘Rutgers’, and ‘Quali T 27’ (henceforth “Quali T”) accumulated more relative shoot biomass than LA0716. Modern and traditional tomatoes had significantly greater relative root biomass than wild tomato (Fig. 1b) (F = 13.01, p = 8.95 × 10^–6^). However, individual varieties did not differ in relative root biomass accumulation (Fig. 1b) (F = 0.91, p = 0.52). Wild tomato also significantly differed from the cultivated varieties regarding its P stress factor (PSF) (F = 7.32, p = 0.001). This metric evaluates the degree to which a certain genotype is negatively affected by P deficiency. Wild tomato had a greater PSF than modern or traditional tomato (Fig. 1c). Phosphorus stress factor also significantly varied among varieties (F = 2.58, p = 0.01). Accession LA1580 had a greater PSF than any other tested variety except for accession LA0716 (Fig. 1c). Visually, stunted growth resulting from P deficiency were minimally observed in modern and traditional tomato as compared to the wild varieties (Fig. 1d).

### Soil phosphorus solubilization changes with tomato domestication

**Figure 2:**
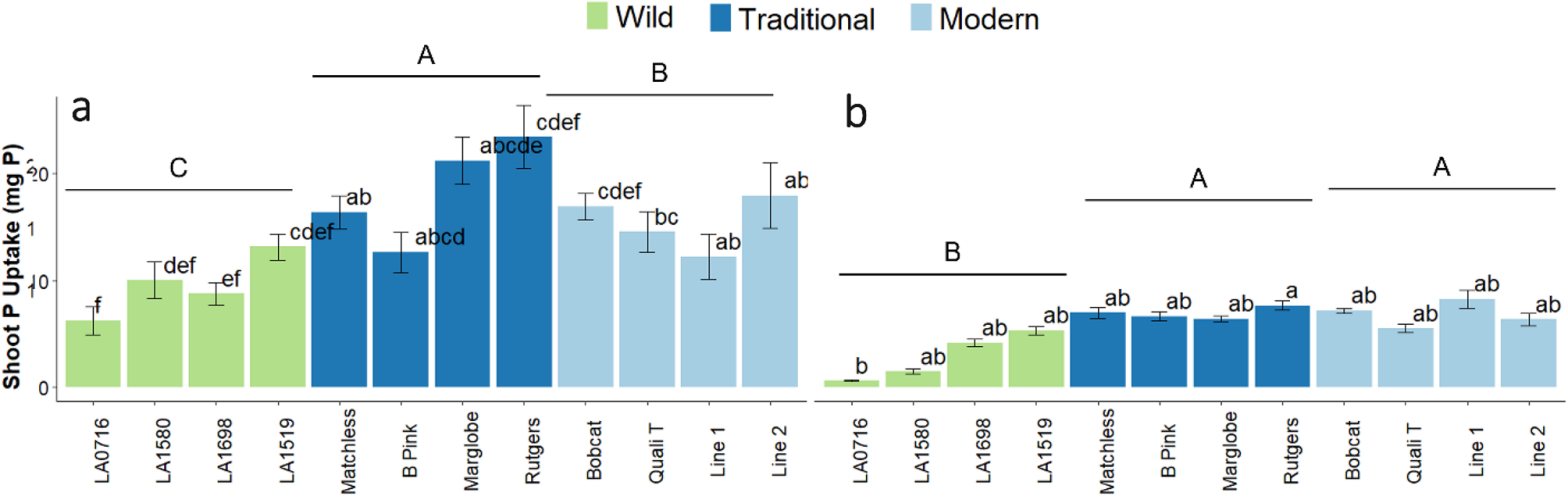
Plant phosphorus (P) uptake (shoot mg P) in tomato across a domestication gradient. (**a**) Shoot P uptake (mg) in the fertilized soil. Presented as mean ± SEM. (**b**) Shoot P uptake (mg) in unfertilized soil. Presented as mean ± SEM.Different colored bars indicate the level of domestication: wild (green), traditional (dark blue), and modern (light blue). An analysis of variance (ANOVA) was performed with a Tukey HSD test for means comparison. Different lowercase letters represent significant differences (p < 0.05) in tomato genotype. Different uppercase letters represent significant differences (p < 0.05) in tomato domestication group. Treatments sharing a common letter are not significantly different. An ANVOA showed differences in shoot P uptake for the Fertilization-Domestication interaction (df = 2, F = 5.223, p = 0.006) and for the Fertilization-Domestication-Variety interaction (df = 18, F = 3.574, p = 4.00 × 10^−6^).

P uptake (shoot mg P) showed a significant interaction effect between fertilization treatment, domestication group, and tomato variety (F = 5.3.57, p = 4.00 × 10^–6^). In fertilized soil, traditional tomato had the greatest P uptake, followed by modern tomato, and wild tomato (Fig. 1). ‘Rutgers’ and ‘Marglobe’ had greater P uptake compared to ‘B Pink’, Line 1, and every wild tomato variety (Fig. 2a). In unfertilized soil, wild tomato had less P uptake than traditional and modern tomato, and Line 1 had a greater P uptake than LA0716. Phosphorus uptake difference from the fertilized treatment to the unfertilized treatment varied because of domestication (F = 5.31, p = 0.006) and variety (F = 2.92, p = 0.005) (Fig. 2b).

**Figure 3:**
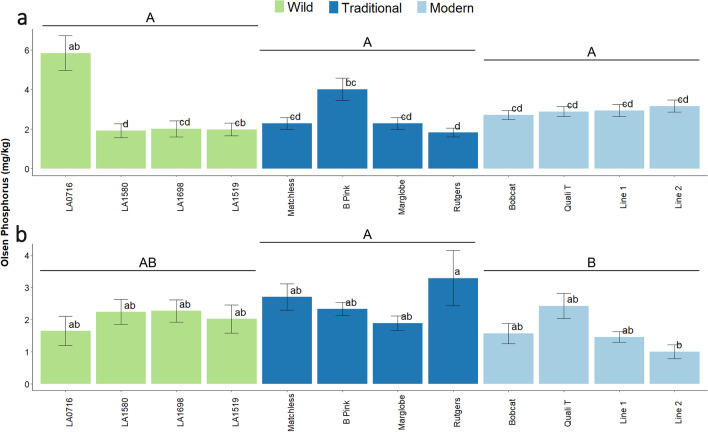
Bulk soil Olsen phosphorus concentration (mg/kg) in tomato across a domestication gradient. (**a**) Olsen phosphorus concentration in fertilized soil. Presented as mean ± SEM. (**b**) Olsen phosphorus concentration in unfertilized soil. Presented as mean ± SEM. An analysis of variance (ANOVA) was performed with a Tukey HSD test for means comparison. While fertilization increased the Olsen-P concentration (p < 0.05), there was no significant difference in Olsen P concentration because of domestication or variety (p > 0.05). Treatments sharing a common letter are not significantly different. Different colored bars indicate the level of domestication: wild (green), traditional (dark blue), and modern (light blue). An analysis of variance (ANOVA) was performed with a Tukey HSD test for means comparison. Different lowercase letters represent significant differences (p < 0.05) in tomato genotype. Different uppercase letters represent significant differences (p < 0.05) in tomato domestication group. Treatments sharing a common letter are not significantly different. An ANVOA showed differences in shoot P uptake for the Fertilization-Domestication interaction (df = 2, F = 5.073, p = 0.007) and for the Fertilization-Domestication-Variety interaction (df = 18, F = 4.996, p = 2.17 × 10^−9^).

There was a significant interaction effect in Olsen-P levels as a function of fertilization, tomato domestication, and variety (F = 5.00, p = 2.17 × 10^–9^). In fertilized soil, tomato domestication groups did not significantly vary in soil Olsen-P levels. Accession LA0716 had greater Olsen-P concentration than every accession except ‘B Pink’ in fertilized soil (Fig. 3a). Regarding soil P levels in unfertilized soil, Olsen-P concentration was higher in traditional compared to modern tomato, and in accession ‘Rutgers’ compared to Line 2 (Fig. 3b).

### Changes in microbial community structure among domestication groups

The rhizosphere microbial community structure across all domestication groups changed with domestication (F = 2.23, p = 0.004), fertilization (F = 18.19, p = 0.001), and variety (F = 51, p = 0.002) (Supplemental Fig. 1). Pairwise adonis testing indicated that wild tomato had a significantly different community structure than modern tomato (p_adj_ = 0.015) and traditional (p_adj_ = 0.03) tomato. However, the community structure did not statistically differ between traditional tomato and modern tomato (p_adj_ = 0.64). Accession LA0716 had a significantly different rhizosphere community structure than most tested accessions (LA1580, LA1519, ‘B Pink’, ‘Rutgers’, ‘Bobcat’, ‘Quali T’, Line 1, Line 2) (Supplemental Fig. 1).

**Figure 4:**
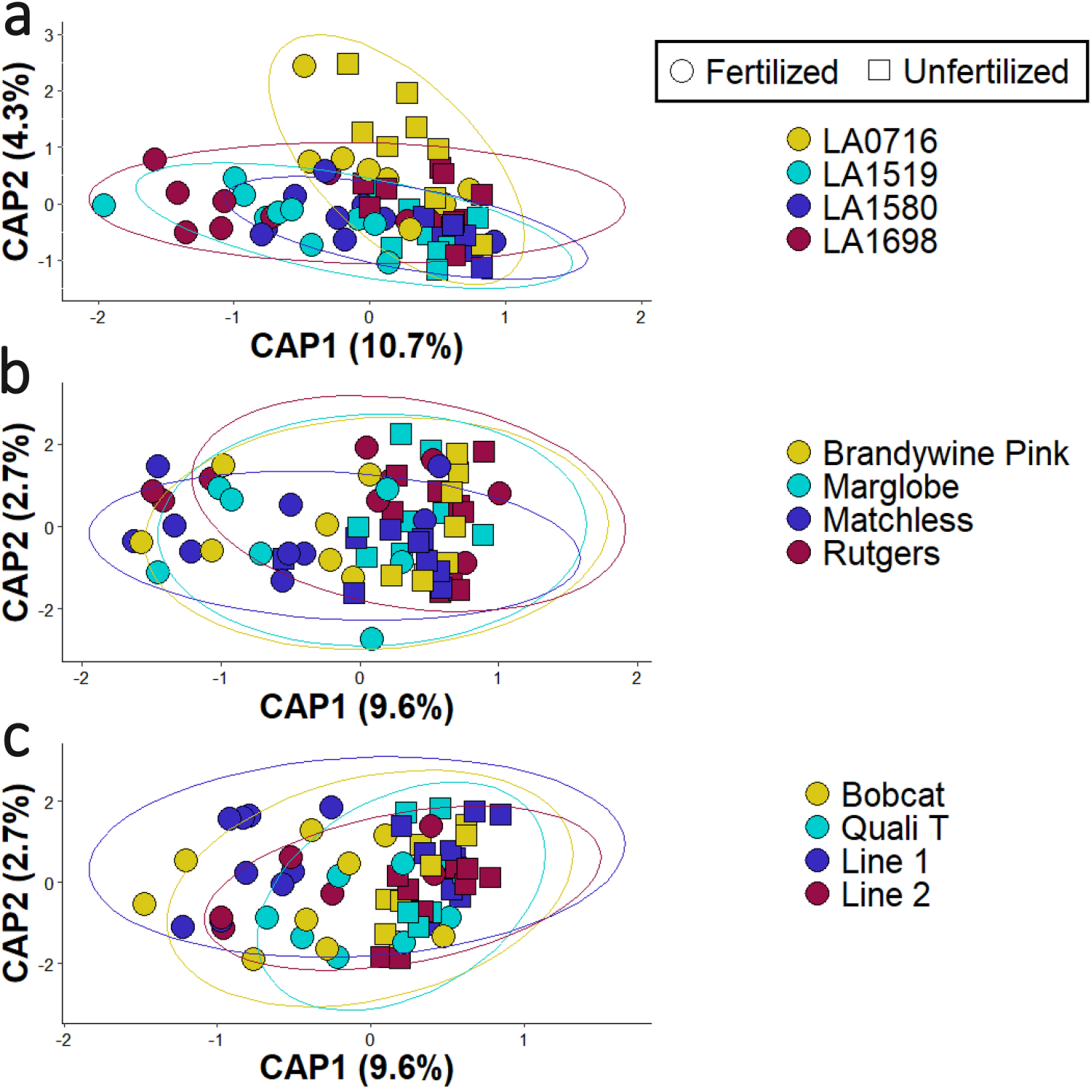
Distance-based redundancy analysis (db-RDA) showing clustering based on Bray–Curtis dissimilarity of the bacterial community structure in the rhizosphere of different tomato domestication groups. (**a**) Wild tomato showed separation based on fertilization treatment (R^2^ = 0.082, df = 1, F = 6.449, p = 0.001) and variety (R^2^ = 0.083, df = 3, F = 2.170, p = 0.001). (**b**) Traditional tomato separated with fertilizer treatment (R^2^ = 0.084, df = 1, F = 6.054, p = 0.001) but not variety (R^2^ = 0.047, df = 3, F = 1.125, p = 0.289). (**c**) Modern tomato separated with fertilizer treatment (R^2^ = 0.108, df = 1, F = 7.5488, p = 0.001) but not variety (R^2^ = 0.045, df = 3, F = 1.06, p = 0.34). The ellipses and colors indicate the different tomato varieties: LA0716, ‘Brandywine Pink’, ‘Bobcat’ (yellow); LA1519, ‘Marglobe’, ‘Quali T’ (light blue); LA1580, ‘Matchless’, Line 1 (dark blue); LA1698, ‘Rutgers’, V8053 (red). The shapes represent the fertilization treatment: fertilized (circle) and unfertilized (square).

Within each domestication group, the bacterial community structure significantly changed with fertilization as explained by the distance-based redundancy analysis (db-RDA), with 15%, 12.3%, and 12.3% variation explained by the varietal and fertilization factors for wild (F = 6.45, p = 0.001), traditional (F = 6.05, p = 0.001), and modern (F = 7.55, p = 0.001) tomato, respectively (Fig. 4). The first two axes of the db-RDAs explained 28.8%, 19.6%, and 18.7% of the variance explained by a PCoA for wild, traditional, and modern tomato, respectively (Supplemental Fig. 2). However, within the wild tomato rhizosphere, the bacterial community structure varied not only with the fertilization treatment, but also by tomato variety (F = 2.17, p = 0.001) (Fig. 4a). Accession LA0716 had a different microbial community structure than did every other wild tomato accession (Fig. 4a). Tomato variety did not change the bacteria community composition within the traditional (F = 1.12, p = 0.29) (Fig. 4b) or modern (F = 1.06, p = 0.34) groups (Fig. 4c). Although beta diversity—as measured by bacterial community composition—varied significantly with tomato domestication groups, Shannon’s alpha diversity did not significantly change as a function of tomato domestication (F = 0.67, p = 0.51) (Supplemental Fig. 3). This measure of alpha diversity only changed with fertilization; fertilized soil increased Shannon’s diversity compared to unfertilized soil (F = 6.53, p = 0.01) (Supplemental Fig. 3).

A differential abundance analysis revealed differences in rhizosphere bacterial abundance among domestication groups as assessed by the log-twofold change in abundance data. Three species [(*Rhizorhapis suberifaciens* (p_adj_ = 8.54 × 10^–30^), *Microcoleus* sp. PCC 7113 (p_adj_ = 0.01), *Rhizobacter gummiphilus* (p_adj_ = 0.001)] were more abundant in the wild tomato rhizosphere compared to the traditional tomato rhizosphere (Table 1). According to PICRUSt2 analysis, two of these bacteria more abundant in the wild group were putative P decomposers (*Rhizorhapis suberifaciens, Rhizobacter gummiphilus*). Ten species changed in abundance between the wild and modern tomato groups. Of these ten species, six were enriched in the modern tomato rhizosphere [*Longimicrobium terrae* (p_adj_ = 0.02), *Nitrospira moscoviensis* (p_adj_ = 0.03), *Brevitalea aridisoli* (p_adj_ = 0.05), *Brevitalea deliciosa* (p_adj_ = 0.03), *Nitrospira japonica* (p_adj_ = 0.007), *Vicinamibacter silvestris* (p_adj_ = 0.03)] and four were enriched in the wild tomato rhizosphere [[*Polyangium*] *brachysporum* (p_adj_ = 0.04), *Geitlerinema* sp. PCC 7407 (p_adj_ = 0.04), *Leptolyngbya* sp. O-77 (p_adj_ = 0.04), *Rhizobacter gummiphilus* (p_adj_ = 7.33 × 10^–7^) (Table 1). Of these ten differentially abundant species, four were identified through PICRUSt2 to be P decomposers (*Nitrospira moscoviensis, Nitrospira japonica,* [*Polyangium*] *brachysporum, Rhizobacter gummiphilus*) and five were P solubilizers (*Nitrospira moscoviensis, Nitrospira japonica,* [*Polyangium*] *brachysporum, Rhizobacter gummiphilus, Vicinamibacter silvestris*). There were no differences when comparing the bacterial counts of traditional and modern tomato.

**Table 1:** Differentially abundant bacteria species enriched in different tomato domestication groups.

Taxa	Predicted phosphorus function	Enriched group	Contrast	Log fold change	Adjusted p value
*Rhizobacter gummiphilus*	Decomposition, Solubilization	Wild	Traditional	1.126	1.29 × 10^–3^
	Decomposition, Solubilization	Wild	Modern	1.398	7.33 × 10^–7^
*Rhizohapis suberfaciens*	Decomposition	Wild	Traditional	32.740	8.54 × 10^–30^
*Microcoleus* sp*. PCC 7113*	NA	Wild	Traditional	1.789	1.45 × 10^–2^
*[Polyangium] brachysporum*	Decomposition, Solubilization	Wild	Modern	0.310	4.05 × 10^–2^
*Geitlerinema sp. PCC 7407*	NA	Wild	Modern	1.117	4.05 × 10^–2^
*Leptolyngbya sp. O-77*	NA	Wild	Modern	1.259	4.31 × 10^–2^
*Nitrospira japonica*	Decomposition, Solubilization	Modern	Wild	0.369	6.69 × 10^–3^
*Longimicrobium terrae*	NA	Modern	Wild	0.5602	1.70 × 10^–2^
*Nitrospira moscoviensis*	Decomposition, Solubilization	Modern	Wild	0.476	2.65 × 10^–2^
*Vicinamibacter silvestris*	Solubilization	Modern	Wild	0.356	3.36 × 10^–2^
*Brevitalea deliciosa*	NA	Modern	Wild	0.381	3.36 × 10^–2^
*Brevitalea aridisoli*	NA	Modern	Wild	0.385	4.55 × 10^–2^

A Wald test was used to determine differences. “Taxa” denotes the bacteria species that had a significantly greater abundance in the treatment represented in the “Enriched Group” column compared to the “Contrast” column. “Log Fold Change” shows the log-twofold change in abundance between the “Enriched Group” and “Contrast”. The p-value was adjusted using a false discovery rate (FDR) at 0.05. KEGG orthologs were used to determine the predicted function indicated in the “Predicted Phosphorus Function”. Based on PICRUSt2, bacteria classified into the “Solubilization” group were predicted to contain the pqqC gene (KEGG Ortholog K06137), and those in the “Decomposition” group were predicted to contain the phoA (KEGG Ortholog K01077), phoD (KEGG Ortholog K01113), phoN (KEGG Ortholog K09474), PHO (KEGG Ortholog K01078), or appA (KEGG Ortholog K01093) gene.

**Figure 5:**
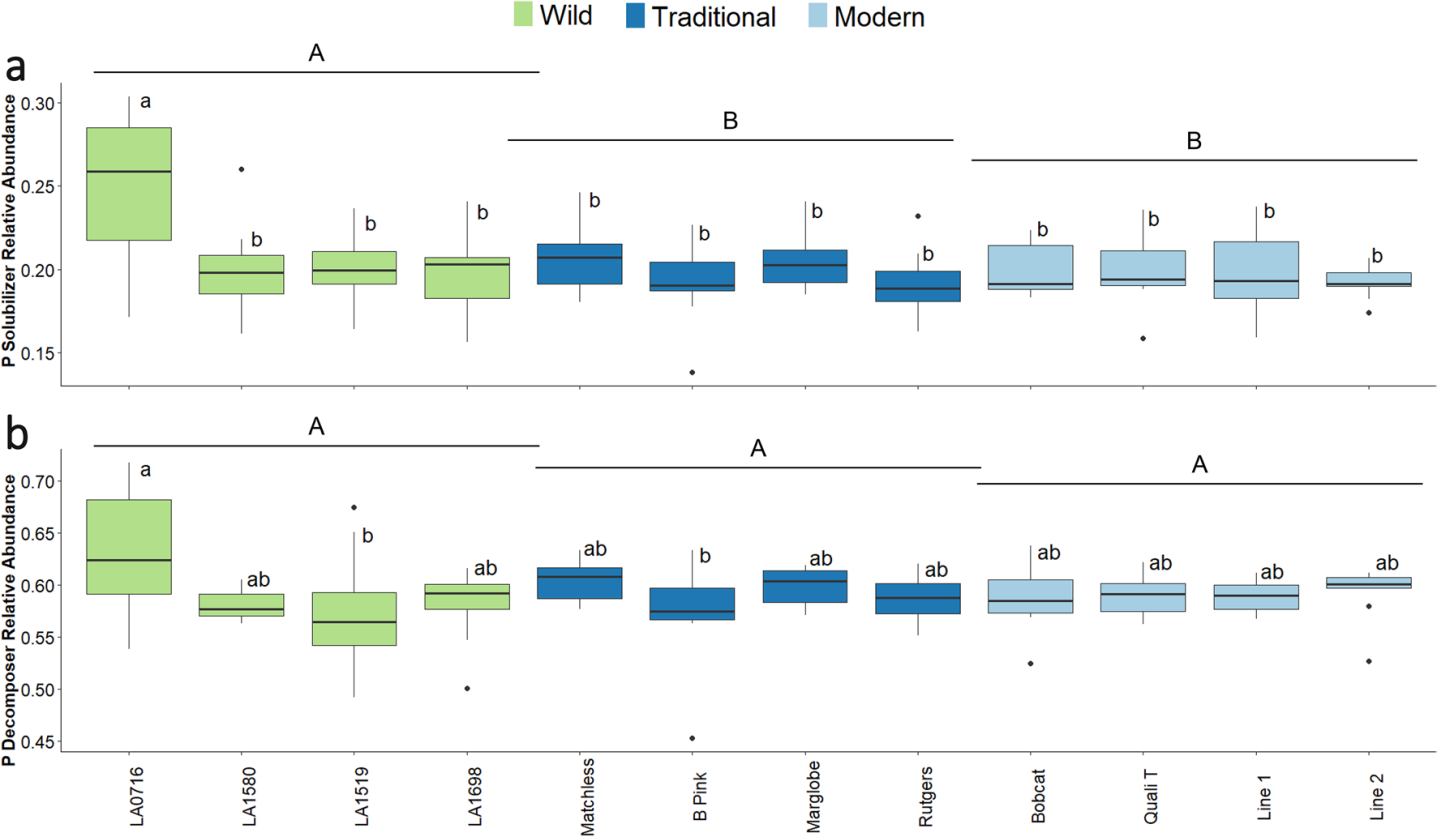
Phosphorus (P) solubilizing and P decomposing bacteria relative abundance in unfertilized soil. Different colored bars indicate the domestication level: wild (green), traditional (dark blue), and modern (light blue). An ANOVA was run with a Tukey HSD test for post-hoc comparison. Different lowercase letters denote significant differences (p < 0.05) for the different varieties. Different uppercase letters denote significant differences (p < 0.05) for the different domestication groups. Treatments sharing a common letter are not significantly different. (**a**) P solubilizing bacteria in unfertilized soil. An ANVOA showed differences in shoot P uptake for Domestication main effect (df = 2, F = 7.25, p = 8.98 × 10^–4^), Fertilization main effect (df = 1, F = 36.47, p = 6.78 × 10^–9^), the Fertilization-Domestication-Variety interaction (df = 18, F = 2.936, p = 1.0 × 10^–4^), but not the Fertilization-Domestication interaction (df = 2, F = 0.114, p = 0.893). (**b**) P decomposing bacteria in unfertilized soil. An ANVOA showed differences in shoot P uptake for Fertilization main effect (df = 1, F = 20.306, p = 1.09 × 10^–5^), the Fertilization-Domestication-Variety interaction (df = 18, F = 2.178, p = 0.005), but not the Fertilization-Domestication interaction (df = 2, F = 0.202, p = 0.817) or Domestication main effect (df = 2, F = 0.097, p = 0.908).

Phosphorus-solubilizing and P-decomposing bacteria functional relative abundance (i.e., proportionate composition of PICRUSt2-determined P-solubilizing and P-decomposing bacteria to the whole bacterial population) were examined to further elucidate differences in microbial composition because of domestication. There was a significant interaction effect between fertilization, tomato domestication group, and variety (F = 2.94, p = 1.1 × 10^–4^). The wild tomato rhizosphere had a greater relative abundance of PICRUSt2-determined P solubilizing bacteria compared to traditional and modern tomatoes in unfertilized soil (Fig. 5a). Accession LA0716 had a greater relative abundance of P solubilizers compared to any other accession (Fig. 5a). While domestication did not change the relative abundance of P decomposing bacteria in the rhizosphere (F = 0.10, p = 0.91), tomato variety did have an effect (F = 2.18, p = 0.005) (Fig. 5b). Phosphorus decomposer relative abundance was higher in the rhizosphere of accession LA0716 compared to accession LA1698 and ‘B Pink’ (Fig. 5b). However, when fertilizer was applied, these differences diminished. In fertilized soil, neither domestication nor tomato variety changed the P solubilizer (Supplemental Fig. 4) or decomposer (Supplemental Fig. 4) relative abundance.

### Crop domestication altered rhizosphere bacteria functions and community dynamics

We ran a comprehensive analysis of PICRUSt2 data to elucidate the genes that may be responsible for the interactions occurring between the tested tomatoes and rhizosphere bacteria in unfertilized soil. We found that some bacteria did not change in abundance among the different domestication groups (Supplemental Table 2). Other bacteria potentially possessing enzymes with different functions (e.g., antibiotic, chelation, P decomposition) were significantly more highly associated with wild tomato roots as compared to modern or traditional tomato (Table 2). These bacteria contain genes that can encode for enzymes that are chelating agents (glycine dehydrogenase, salicylate synthetase), have antifungal (phloroglucinol synthase, iturin family lipopeptide synthetase D) and antibiotic (AHBA synthesis associated protein, 2-amino-4-deoxychorismate synthase) properties, decompose nitrogen (N) (urease alpha subunit), reduce nitrate (nitrite reductase), and promote denitrification (nitrate reductase, nitric oxide reductase) (Table 2). Only one predicted enzyme, isochorismatase, was in lower abundance in the rhizosphere of wild tomato (Table 2). Rather, isochorismatase-harboring bacteria were more highly associated in the rhizosphere of traditional tomato compared to wild or modern tomato (Table 2). There were also predicted gene functions that did not change in abundance as a function of domestication (biocontrol, root growth, chelation, carbon decomposition, denitrification, P decomposition, P solubilization, N fixation) (Supplemental Table 2).

**Table 2:** Varied relative abundance of bacteria with predictive functions among tomatoes across a domestication gradient in unfertilized soil.

Enzyme (Gene)	Function (KEGG Ortholog)	F	Adj. p	Relative abundance		
				Modern	Traditional	Wild
Glycine dehydrogenase (*hcnA*)	Chelation^26^ (K10814)	6.58^L^	0.027	0.053 ± 0.001 b	0.054 ± 0.002 b	0.067 ± 0.004 a
Phloroglucinol synthase (*phlD*)	Antifungal^27^ (K15431)	6.04^L^	0.040	0.053 ± 0.001 b	0.054 ± 0.002 b	0.066 ± 0.004 a
Iturin family lipopeptide synthetase A (*ituA*)	Antifungal^28^ (K15661)	4.82^L^	0.045	0.055 ± 0.001 b	0.055 ± 0.002 b	0.065 ± 0.004 a
Fengycin family lipopeptide synthetase D (*fenA*)	Antifungal^29^ (K15667)	4.70^L^	0.045	0.065 ± 0.001 b	0.065 ± 0.002 b	0.075 ± 0.004 a
AHBA synthesis associated protein (*rifM*)	Antibiotic^30^ (K16017)	4.71^L^	0.045	0.056 ± 0.001 b	0.056 ± 0.002 b	0.068 ± 0.004 a
2-amino-4-deoxychorismate synthase (*phzE*)	Antibiotic^31^ (K13063)	5.38^L^	0.045	0.063 ± 0.001 b	0.064 ± 0.002 b	0.076 ± 0.005 a
Salicylate synthetase (*mbtI*)	Chelation^32^ (K04781)	4.98^L^	0.045	0.057 ± 0.001 b	0.057 ± 0.002 b	0.067 ± 0.004 a
Isochorismatase (*entB*)	Chelation (EC.3.3.2.1_1)	7.95	0.027	0.029 ± 0.009 b	0.033 ± 0.001 a	0.028 ± 0.001 b
Urease alpha subunit (*ureC*)	Nitrogen decomposition^33^ (K01428)	5.31	0.045	0.376 ± 0.005 b	0.383 ± 0.007 b	0.413 ± 0.013 a
Acid phosphatase (*phoN*)	Phosphorus decomposition^33^ (K09474)	6.65^L^	0.035	0.071 ± 0.005 b	0.071 ± 0.002 b	0.087 ± 0.005 a
Nitrite reductase (NAD(P)H) (*NIT-6*)	Assimilatory nitrate reduction^34^ (K00366)	4.65^L^	0.045	0.053 ± 0.001 b	0.053 ± 0.002 b	0.064 ± 0.004 a
Nitrite reductase (NO forming) (*nirS*)	Denitrification^35^ (K15864)	5.85^L^	0.042	0.057 ± 0.001 b	0.058 ± 0.002 b	0.071 ± 0.004 a
Nitric oxide reductase (*norC*)	Denitrification^36^ (K02305)	4.68^L^	0.045	0.083 ± 0.002 b	0.084 ± 0.003 b	0.098 ± 0.005 a

Bacterial relative abundance (presented as mean ± SEM) in unfertilized soil is shown for each tomato domestication group (Modern, Traditional, Wild). An ANOVA (F and FDR-adjusted p values presented) with Tukey HSD at α = 0.05 was used to determine differences in the relative abundance of rhizosphere bacteria as a function of tomato domestication group. Different letters within each row denote significant differences in the relative abundance of the bacteria with corresponding tested gene. For data that did not have normally distributed residuals, a log-transformation was run (denoted as “^L^” in the F-value column). “Function” represents the predictive function for the bacteria with the corresponding gene listed in the “Enzyme (Gene)” column. This table shows the tested genes that were significantly different (Table S2 contains the tested genes that were non-significantly different).

## Discussion

Rock phosphate, from which phosphorus (P) fertilizers are mined, is a nonrenewable resource. Thus, efforts are needed to enhance P acquisition and recovery. Phosphorus stress responses vary not only among species, but also among different genotypes of the same species. Genotypic variation may, in part, result from domestication, as exemplified by bean (*Phaseolus vulgaris*)^37^ and maize (*Zea mays*)^38^. Araujo et al.^37^ found that wild beans accumulated less biomass than cultivated beans in soils with slightly less than optimal P levels (20 mg/kg P). Further, Perkins and Lynch^38^ identified that a greater number of embryonic roots in domesticated maize compared to wild teosinte contributed to increased P acquisition in domesticated maize. However, studies investigating P acquisition-related traits at different stages of breeding, not just domestication, are lacking. Here, we examined the P acquisition response of wild, traditional, and modern tomato varieties by observing the effects of low P on plant growth and rhizosphere microbial community composition. We found that domestication altered tomato P acquisition traits, but among the traditional and modern varieties, P acquisition responses were similar.

Wild tomatoes may have relied on microbial associations to acquire P and cultured a unique soil microbiome (Supplemental Fig. 1). Bacterial community composition varied with fertilization for all types of tomato, but for wild tomato, there were compositional differences as a result of variety (Fig. 4). Approximately 10% of the total variation in bacterial community structure was explained by fertilization and approximately 3–4% of the variation was explained by the tomato variety grown, suggesting that tomato genotype and P fertilization have small but significant effect on the soil microbiome. Across all tomato varieties in our study, accession LA0716 associated with distinct soil bacteria (Supplemental Fig. 1) and accumulated a greater relative abundance of possible P solubilizing bacteria (Fig. 5). For example, *Rhizobacter gummiphilus*, which possesses the *phoD* gene that contributes to organic-P decomposition, was in greater abundance in the LA0716 rhizosphere. This accession has recently been shown to exude elevated amounts of acid phosphatase in response to P deficiency^39^. Increased phosphatase activity increases organic P decomposition, thereby increasing suitable conditions for soil microbes. Thus, heightened phosphatase exudation of LA0716 may further promote growth of *phoD*-harboring bacteria. Further, LA0716 also proliferates root hair growth in response to a P deficiency^39^, and increased root hair growth is associated with greater root exudation rates and microbial diversity^40^. By forming associations with *phoD*-harboring bacteria, LA0716 prioritizes the strategy of decomposing sparingly available soil P pools. LA0716 is partially insensitive to levels of bioavailable phosphate, as outlined by Demirer et al.^39^, which may explain why this accession uniquely responded to low-P stress compared to not only modern and traditional tomato, but also to other accessions of wild tomato.

As a group, wild tomato accessions had a greater relative abundance of P-solubilizing bacteria (Fig. 5a), even though not every predicted P cycling function was in greater abundance in the wild tomato group (Table 2). This finding is in agreement with Tang et al.^41^ who found that phosphatase exudation was greater in wild compared to domesticated barley. The interactions between roots and soil microbes that facilitate nutrient cycling may be more present in wild tomato and diminished with domestication. Therefore, we suggest that the methods of P recovery employed by tomatoes diverged at the initial event of domestication, but not after subsequent breeding and selection events. Wild tomato appears to rely on microbial P decomposers, but this reliance is not present in the modern and traditional tomato varieties which are more tolerant to P deficiency. However, these bacteria were identified using the predictive tool, PICRUSt2, which uses marker genes to map functions to microbial communities. Thus, although there is no direct confirmation of the functional capabilities of the selected microbes in this study, we can infer the functions which may be associated with bacterial taxa^42^.

Our results indicate that wild tomato form associations with beneficial microbes, compared to either of the domesticated groups, as supported by wild tomato accumulating a high abundance of bacteria with the P-solubilizing, *pqqC* gene. Numerous studies have delineated the benefits to plant growth by P-solubilizing bacteria as a result of their organic acid production^43^, auxin production^44^, pathogen suppression^45^, and ability to dissociate calcium-phosphate bonds^46^. Thus, it is possible that increasing the relative abundance of possible P solubilizing bacteria results in improved plant growth. Our tested wild tomato accessions showcased a high abundance of putatively beneficial bacteria, such as cyanobacteria members (*Geitlerinema* sp. PCC 7407, *Leptolyngbya* sp. O-77, *Microcoleus* sp. PCC 7113), which further supports our hypothesis that wild tomato relies heavily on microbial P scavenging strategies to grow and develop with low P. Afkairin et al.^47^ found that in low-P soil, cyanobacteria additions increased the pool of soluble P across a seven week period; our experiment took place in a similar soil and over a similar time frame. Thus, the presence of these species may increase the pool of bioavailable P. The finding that wild tomato forms more associations with beneficial bacteria (as compared to traditional or modern tomato) is in congruence with past research focusing on the effects of domestication and symbiosis with plant growth promoting soil bacteria. Symbiosis with beneficial, nodule-forming bacteria (*Rhizobium leguminosarum*), for instance, had a greater ability to cause nodulation in wild legumes (*Vicia* spp., *Lathyrus* spp.) compared to cultivated relatives (*Pisum sativum*, *Vicia faba*)^48^. This finding is also supported by Kovacs et al.^17^, who found that wild tomato had greater rhizosphere phospholipid fatty acid content than cultivated tomato, which would suggest a greater absolute microbial abundance in the wild tomato rhizosphere.

This study showed that wild tomato, in addition to interacting with P-solubilizers, highly associated with many other PICRUSt2-predicted beneficial rhizosphere bacteria (Table 2). Wild tomato, for example, had a greater abundance of bacteria that produce siderophores and hydrogen-cyanide (HCN) in unfertilized soil compared to modern and traditional tomato (Table 2). Siderophores and HCN are chelating agents, and bacteria that produce these compounds have been identified to help enhance P solubility in soil systems^26,49^. Thus, the tested accessions of wild tomato may strategize soil P recovery by forming associations with bacteria that target P solubilization through diverse mechanisms (i.e., Fe–P chelation, Ca-P solubilization, organic-P decomposition). Many of the bacteria that were in greater abundances in the wild tomato rhizosphere compared to the traditional and modern tomato rhizosphere were predicted to be involved in biocontrol (i.e., produce enzymes with antibiotic and antifungal properties) (Table 2). Production of antifungal and antibacterial properties helps to support plant growth indirectly by mitigating or preventing negative effects of phytopathogens^50^. Therefore, the ability of wild tomato to strengthen associations with diverse soil bacteria supports the conclusion that reliance on microbial associations has diminished at domestication but has not been further affected. This conclusion is supported by Cordovez et al.^19^ who investigated variation between wild and domesticated crop rhizosphere microbiomes. They found that not only are there differences in the soil microbiome of wild and cultivated tomato, but these differences are also amplified across planting cycles^19^. Yet, this trend may not be true across all crop species. Brisson et al.^51^ found that a wild accession and a cultivated accession of maize both harbored phosphate solubilizing bacteria in the rhizosphere and had a similar root exudate response to P deficiency. However, because the researchers tested only one accession of each domestication group^51^, and because maize has high genetic diversity^52^, it is difficult to conclude whether or not this trait has been conserved through domestication and breeding. This contrasting result may also suggest that there is variation among different species in P acquisition as a function of domestication.

Wild tomato had a greater abundance of PICRUSt2-predicted P solubilizers, P decomposers, and chelating agent producers than domesticated counterparts, which may be explained by changes in root exudate profiles that occur with domestication. A recent study by Sun et al.^53^ illustrated how the varied root exudate profiles in wild and domesticated rice (*Oryza* spp.) caused a shift in bacterial chemotaxis systems in the rhizosphere and concluded that wild rice has co-evolved with microbes to adapt to harsher environments. Similarly, in our studies the exudate profile of tomato may explain how it interacts with the rhizosphere microbiome. In their study on root exudates of P deficient crops, Neumann and Römheld^54^ found that hydroponic-grown tomato (cv. Moneymaker) highly exudes protons when in low P conditions, reducing the pH from neutral (7.0–7.5) to acidic (4.7). The ‘Moneymaker’ cultivar is a domesticated variety of tomato, and it is possible that wild tomato may not be exuding protons to the same degree as domesticated tomato. In calcareous soils, rhizosphere pH has been shown to increase near wild plants^55^. Therefore, because rhizosphere acidification through proton exudation may increase bioavailable P levels^56^, traditional and modern tomato varieties may be prioritizing root exudation as a strategy to recover P from soils. Because root exudates impose strong selection power on the development of the microbiome^57^, the varied root exudate profiles of the different tomato domestication groups in our study may be, in part, responsible for the changes observed in the microbial community composition. Therefore, exploration of root exudation provides possible avenues for future studies on the interactions between plant breeding events and the soil microbiome.

The possible changed exudate profiles may have caused a different microbial community composition in our tested accessions. Although the rhizosphere microbiome contained a high relative abundance of PICRUSt2-inferred beneficial bacteria in the wild tomato soil, modern tomato had a greater abundance of certain beneficial microbes. When comparing modern to wild tomato, modern tomato had a greater abundance of *Nitrospira moscoviensis* (Fig. 5b)*,* which facilitates carbon-P and nitrogen-P conversions^58^. However, when comparing modern to traditional tomato, there were no differences in the community structure or specific bacterial species abundances. In our studies, modern and traditional tomatoes had similar responses to P deficiency, with the exception of Olsen-P. Modern tomato had less Olsen-P than traditional tomato in the low P treatment (Fig. 3b). However, Recena et al.^59^ showed that in low P soil, Olsen-P may not accurately predict plant P uptake. Therefore, although Olsen P varied with these two domesticated groups, this result on its own may not indicate that they differ in P efficiency or in their strategies to acquire P. Both traditional and modern tomatoes had greater relative shoot biomass (Fig. 1a), relative root biomass (Fig. 1b), lower P stress factor (PSF) (Fig. 1c), and greater shoot P uptake (Fig. 2a) than wild tomato. These differences illustrate the varied magnitude of impact P deficiency had on each tested domestication group. PSF was greater in wild compared to traditional or modern tomato (Fig. 1c), which suggests that wild tomato may have less of an ability to efficiently acquire or internally utilize P^60^, and therefore, cultivated tomatoes are more tolerant to P stress than wild tomato. This varied degree of tolerance in the tested domestication groups regardless of the rhizosphere microbial composition may be a result of crop improvements at domestication. For example, in a recent study of domesticated and wild tomato, it was found that the functions of soil bacteria in domesticated tomato were more highly affiliated with xenobiotic biodegradation and metabolism than wild tomato^61^. Therefore, although wild tomato may be accumulating a high concentration of P solubilizing bacteria—leading to potentially more bioavailable P—domesticated tomato harbor bacteria carrying out other plant-beneficial functions that may promote growth in nutrient-deplete conditions. These observed variations indicate that while reliance on microbial associations may be lessened at the point of domestication, domesticated varieties employ other strategies of soil P recovery.

Both cultivated groups had fewer PICRUSt2-predicted P solubilizers in the rhizosphere of low P soil (Fig. 5a), suggesting that domesticated tomato may be utilizing intrinsic P acquisition strategies that are different from those employed by wild tomato. These strategies may include efficient translocation of P within plant tissue, as shown by P-utilization efficient bean^62^ and lettuce^63^. Further, the larger root systems may lead to greater soil aeration and root exudation rates^40^. It is also possible that our tested modern and traditional tomatoes may be efficient in internal P sensing mechanisms. For example, regardless of P acquisition efficiency, *Banksia* spp.is highly P efficient because of its ability to effectively remobilize P from older and senescing tissue^64^. Rather than relying on *pqqC-*harboring bacteria, domesticated tomato may be associating with other beneficial bacterial symbionts. For example, modern tomato had a greater abundance of the PICRUSt2-predicted P decomposer, *Nitrospira japonica* (Table 1). *Nitrospira,* a genus of bacteria involved in P cycling from its enzymatic activity and its ability to alter N and C soil content^65^, has been shown to be present in the rhizosphere of domesticated tomato and is abundant near roots when resources are scarce^66^. Although this present study did not investigate symbiosis with arbuscular mycorrhizal (AM) fungi, this strategy is likely not what is enhancing low-P tolerance of our modern and traditional varieties. As shown by Bryla et al.^67^, cultivated tomato (*Solanum lycopersicum* ‘large cherry’) is less responsive to colonization by AM fungi as compared to wild tomato (*S. lycopersicum* var. *cerasiforme*). Further, when P is supplied, domesticated crops decrease symbiosis with AM fungi to a greater degree than wild relatives^68^.

The rhizosphere microbiome was similar for traditional and modern tomato. Of the cultivated tomato groups, there were no differences in predicted P solubilizer or P decomposer abundance (Fig. 5, Supplemental Fig. 3), and a differential abundance analysis showed that there were no bacterial species that changed in abundance between traditional and modern tomato. The tested traditional tomato accessions were developed and grown before the Green Revolution (late 1800s to early 1900s), whereas the modern tomato accessions are either grown today or are experimental lines for incorporation into future tomato markets. Overarching tomato breeding goals do not include nutrient use efficiency, but rather involve increasing productivity, disease resistance, and fruit health value^69^. Complex traits, such as low P tolerance, were likely indirectly selected through the tomato breeding process. Thus, cultivated tomato varieties respond similarly to a P deficit regardless of being developed in the 1800s or 2000s. However, because this study took place over a short time (approximately 3 months), the changes observed may not truly reflect the changes that may have occurred during the entirety of plant domestication or across a plant’s reproductive development. Therefore, future studies would benefit from identifying changes in the rhizosphere across a tomato’s growth stages.

In summary, our results suggest that wild tomatoes associate more preferably with bacteria that have beneficial functions, perhaps due to a greater reliance on microbes for P acquisition. The differences seen in wild tomatoes compared to the cultivated relatives indicate that there are changes in P acquisition efficiency due to domestication. Our results suggest that wild tomatoes, especially accession LA0716, rely heavily on associations with beneficial P bacteria to grow and develop under P deficiency. Conversely, modern and traditional tomato may be using strategies separate from microbial symbiosis to mitigate P deficiency symptoms. Although the tested domesticated tomato varieties were more tolerant to P stress, they were still negatively impacted by P deficiency as shown by reduced biomass and shoot P uptake. Future studies may be able to increase P acquisition efficiency by combining the divergent P acquisition methods observed here between cultivated and wild tomato and emphasizing those strategies diminished at domestication. Ultimately, the approach used in this study could potentially be applied to other crops to further reduce our reliance on non-renewable rock phosphate.

## Methods

### Plant material selection

Twelve accessions were selected that represent tomato (*Solanum* spp.) across three domestication groups: modern, traditional, and wild (Supplemental Table 1). Modern tomato was defined as commercial cultivars grown currently (‘Bobcat’, ‘Quali T’) and experimental lines for incorporation into the commercial tomato market (Line 1, Line 2). Modern tomato accessions were donated by Syngenta (Delaware, United States) and are determinate, processing tomatoes grown in the San Joaquin Valley in California. Traditional tomato were commercial varieties developed before the Green Revolution and these seeds were retrieved from the W. Atlee Burpee Co. (‘Rutgers’ and ‘B Pink’) and Victory Seed Co. (‘Matchless’ and ‘Marglobe’). The traditional varieties used are semi-determinate to indeterminate and are grown for fresh market. Wild tomato accessions were obtained from the Tomato Genetics Resources Center (TGRC) at the University of California Davis. The wild tomato species used included *Solanum pennellii* (LA0716), *S. pimpinellifolium* (LA1580), and *S. lycopersicum* var. *cerasiforme* (LA1519, LA1698). *S. pennellii* is adapted to grow in dry and rocky conditions with poor nutrient availability^39^, and crosses of this species into cultivated tomato have led to improved abiotic stress tolerance^70^. *S. pimpinellifolium* is the wild progenitor of cultivated tomato, and is a valuable resource in tomato breeding due to its stress tolerance^71^. *S. cerasiforme* originated from *S. pimpinellifolium* and migrated to Mesoamerica which may have resulted in reduced polymorphisms and heterozygosity^72^.

### Soil collection

To induce P scavenging responses from plants, soils were sought out that had low bioavailable P and a total P concentrations that would adequately supply P to plants if P were in an available form. Soil with this characteristic was identified at the Colorado State University (CSU) Agricultural Research, Development, and Education Center (ARDEC) in Fort Collins, CO. Soil was collected at a depth of 5–20 cm and was subsequently mixed with sand (Quikrete Play Sand, Georgia, United States) at a 1:1 by volume ratio to further dilute the P concentration. Water holding capacity was determined following the method of Afkairin et al.^47^. Bioavailable P was measured using the Olsen-P bicarbonate extraction method^73^. The diluted substrate had a low Olsen extractable P concentration (2.6 mg/kg) and greater total P concentration (371 mg/kg) (Table 3). The physical and chemical characteristics were analyzed by Ward Laboratories (Kearney, NE) (Table 3).

**Table 3:** Physical and chemical characteristics of soil and sand substrate. Agricultural soil was mixed at a 1:1 by volume ratio with sand.

Substrate characteristic	Value
Soil pH	8.4
Soluble salts (mmho/cm)	0.47
Water holding capacity (%)	12.0
Organic matter LOI (%)	0.9
Sum of cations (me/100 g)	19.3
Nitrate–N (mg/kg)	3.9
Potassium (mg/kg)	130
Calcium (mg/kg)	3274
Magnesium (mg/kg)	285
Sodium (mg/kg)	58
Zinc (mg/kg)	1.04
Iron (mg/kg)	8.3
Manganese (mg/kg)	3.2
Copper (mg/kg)	0.38
Olsen-phosphorus (mg/kg)	2.6
Total phosphorus (mg/kg)	371

### Greenhouse study conditions

Tomato seeds were surface sterilized with 3% sodium hypochlorite for 30 min followed by five rinses of sterile distilled water, as recommended by the TGRC to readily germinate cultivated and related tomato seeds. The seeds were subsequently placed on moistened filter paper until radicle emergence. At radicle emergence, emerged seedlings were transplanted to a commercial potting mix comprised of sphagnum peat moss, pine bark, and perlite (PromixBK, Québec, Canada) until cotyledons fully expanded. After cotyledon expansion, seedlings were transplanted to pots (117 cm^2^ surface area, 13 cm height) filled with a 1:1 by volume mixture of sand and soil.

Seedlings were left to establish in the soil and sand mixture for seven days before fertilization. There were two fertilizer treatments: a treatment fertilized to P sufficiency (high P) and an unfertilized treatment (low P). Because the soil mixture was deficient in nitrogen (N) (3.9 mg/kg) (Table 3), Environmentally Smart Nitrogen (ESN) (44-0-0) was applied at a rate equivalent to 118 kg/ha N to all treatments. Triple superphosphate (TSP) (0-46-0) was applied to plants at rates equivalent to 163 kg/ha P_2_O_5_ in the P sufficiency treatment. The rates of N and P fertilization were designed for optimum tomato yield based on preliminary soil tests (Ward Laboratories). Plants were arranged in a completely randomized design as determined by a research randomizer software^74^. There were ten replicates per treatment for a total of 240 plants.

Tomato plants were grown from June to August 2022 in a greenhouse at Colorado State University (CSU), Fort Collins, CO (40.572, − 105.081). Temperatures ranged from 18 to 21 °C during the day and 17 to 20 °C during the night, with a photoperiod of 16 h.

### Biomass and soil sampling

Each pot represents an experimental unit. Therefore, for biomass and soil collection, one sample of shoot biomass, root biomass, bulk soil, and rhizosphere soil was collected per pot (N = 240). Because plant interactions with microbial inoculants change at the flowering stage^75^, samples were harvested during the vegetative phase so as not to conflate observed differences in the rhizosphere microbiome being caused by growth stage as opposed to genotype. To ensure all 12 varieties of tomato were harvested at the same developmental stage, harvest occurred at the end of the vegetative stage (8 weeks), and if any flowers showed signs of developing, they were removed. Plants were removed from pots and gently shaken to remove any loose soil. Soil that did not adhere to the roots was considered bulk soil and was collected for nutrient analysis. Remaining soil adhering to the roots was considered rhizosphere soil^74^ and was removed from the root by gently scraping roots with surface sterilized (70% EtOH) polypropylene spatulas. Rhizosphere soil was collected in a sterile 15 mL falcon tube and stored in a − 80 °C freezer until DNA extraction.

After rhizosphere soil was collected, the roots were washed with water to remove any remaining soil. Roots were separated from shoots, and both were placed in a drying oven set at 70 °C for 4 days. Dry root and shoot biomass were recorded. To account for natural variation in biomass accumulation among tomato genotypes, relative dry mass (RDM) and P stress factor (PSF) were calculated following the method of Bera et al.^76^:

$$RDM (\%)= \frac{{DM}_{P-}}{{DM}_{P+}}\times 100$$

$$PSF \left(\%\right)= \frac{{DM}_{P+}-{DM}_{P-}}{{DM}_{P-}}$$

where “${DM}_{P-}$” is dry mass in the unfertilized treatment, and “${DM}_{P+}$” is dry mass in the fertilized treatment. Expressing measurements in relative terms allowed for screening of P-efficiency traits for many genotypes which may express broad morphological variation naturally. Thus, a genotype with high RDM and low PSF would suggest that it is capable of tolerating P stress.

### Nutrient analysis

Bulk soil was air-dried for four days and subsequently processed through a 2 mm sieve. Bulk soil Olsen-P content, representing bioavailable P, was measured^73^. Individual shoot samples were ground to a fine powder and subsequently underwent a concentrated nitric acid digest following the method of Ippolito and Barbarick^77^. Total P concentration in tomato shoots were analyzed using inductively coupled plasma-atomic emission spectroscopy.

### DNA extraction and 16S rRNA amplicon sequencing

Rhizosphere soil samples were weighed to 0.25 g and total genomic DNA was extracted from these samples using the DNeasy Power Soil DNA isolation kit (Qiagen, Hilden, Germany) according to the manufacturer’s instructions. DNA concentration (ng/µL) was quantified using a Qubit Fluorometer (Thermo Scientific, Illinois, United States).

Following DNA extraction, the bacterial 16S ribosomal RNA (rRNA) gene was amplified via polymerase chain reaction (PCR). DNA extracts were diluted at a rate of 1:20, as determined by the Qubit concentrations, with nuclease-free water. For every 4 µL of diluted DNA, 36 µL of master reaction mix was added, which contained 20 µL of Phusion HSII master mix (Thermo Scientific, Illinois, United States), 14.4 µL of nuclease free water, 0.8 µL of forward primer (27F Bacterial Mn, 5’—TTTCTGTTGGTGCTGATATTGC AGRGTTYGATYMTGGCTCAG—3’), and 0.8 µL of reverse primer (1492 Universal Mn, 5’—ACTTGCCTGTCGCTCTATCTTC TACCTTGTTACGACTT—3’). This mixture was amplified under the following conditions: 98 °C for 30 s (1 cycle); 98 °C for 15 s, 50 °C for 15 s, 72 °C for 60 s (25 cycles); and 72 °C for 5 min (1 cycle).

The PCR products were removed from the thermocycler and were purified using paramagnetic beads (AMPure XP beads, Beckman Coulter, Brea, CA). Briefly, the paramagnetic beads selectively bound to the nucleic acids in our sample, and the adhered DNA was rinsed for 30 s twice in 70% ethanol and eluted. The DNA in the purified PCR products was quantified fluorometrically and then diluted with nuclease-free water to reach a DNA concentration of 4 ng/µL. This diluted and purified PCR product was barcoded using 1 µL of sample-specific barcode per sample from the PCR Barcoding Expansion kit (Oxford Nanopore, Oxford, United Kingdom). In a new PCR plate, the 1 µL of sample-specific barcode, and 5 µL of the purified PCR product were added to 25 µL of Phusion HSII master mix and 19 µL of nuclease-free water. This mixture was amplified in a thermocycler under the following conditions: 98 °C for 30 s (1 cycle); 98 °C for 15 s, 62 °C for 15 s, 72 °C for 60 s (15 cycles); and 72 °C for 5 min (1 cycle).

PCR products were volumetrically pooled and were again purified using paramagnetic beads. The samples underwent ligation using the Ligation Sequencing Kit V14 (SQK-LSK114) (Oxford Nanopore Technologies, Oxford, United Kingdom) following manufacturer’s instructions. DNA was quantified and adjusted to 20 ng/µL DNA. To prepare the minION flow cell (Oxford Nanopore Technologies, Oxford, United Kingdom) for sequencing, air was removed with a pipette and the flow cell was primed with a flush buffer. The library (50 mM) was added to the sample port in the flow cell. Sequencing data were collected over a period of 48 h using MinKNOW (Oxford Nanopore Technologies, Oxford, United Kingdom). Resulting signal data were processed (base call, demultiplex) through Guppy basecaller and were filtered to a 70 q score. Taxonomic abundance profiles were generated using Emu, a microbial profiling software^78^. Data were then filtered to retain samples with greater than 10,000 reads.

KEGG orthologs that were used to determine predictive functions were identified using PICRUSt2^79^ to map the proportion of the microbial community to the gene of interest (Supplemental Table 3). Phosphorus decomposing bacteria were those that have been identified to have at least one of the following genes: *appA* (acid phosphatase)*, phoA* (alkaline phosphatase)*, phoD* (alkaline phosphatase D)*,* or *phoN* (class A acid phosphatase). Phosphorus solubilizing bacteria were those that possessed the *pqqC* gene (pyrroloquiniline-quinone synthase).

### Statistical analysis

RStudio version 4.1.2 was used for all statistical analyses. A nested analysis of variance (ANOVA), Y ~ Fertilization*Domestication /Variety, was used to determine differences in Y (PSF, shoot P uptake, Olsen-P, P solubilizer abundance, P decomposer abundance) at an α = 0.05. “Fertilization” indicates the fertilizer treatment (unfertilized, fertilized to P sufficiency). “Variety” represents the 12 tomato accessions (LA0716, LA1580, LA1519, LA1698, ‘B Pink’, ‘Marglobe’, ‘Matchless’, ‘Rutgers’, ‘Quali T’, ‘Bobcat’, Line1, Line 2), and “Domestication” denotes the 3 domestication groups (wild, traditional, modern). Fertilization was incorporated into the calculation of relative shoot biomass, relative root biomass, and difference in shoot P, so “Fertilization” was removed from models testing those metrics. According to the residuals-leverage plot, there appeared to be an outlier present in the RDM and Olsen-P models. Using Studentized residuals, a Bonferroni outlier test determined if there was a mean-shift outlier present in these data. Replicate 8 of accession LA1580 was removed from the RDM calculation (Bonferroni-adjusted p = 3.5 × 10^−40^) and replicate 8 of fertilized accession LA0716 was removed from the Olsen-P calculation (Bonferroni-adjusted p = 5.8 × 10^−122^). All the above analyses used a Tukey HSD test for mean comparison at an α = 0.05.

A permutational analysis of variance (PERMANOVA) was run for each domestication group to examine differences in microbial community structure (Distance ~ Fertilization*Variety). “Distance” refers to the Bray–Curtis dissimilarity, which is a method to quantify distance and ordinate samples onto axes by their relation to reference points^80^. This model was run for each domestication group (wild, traditional, modern). To examine effects across all tested accessions, a nested PERMANVOA was used (Distance ~ Fertilization*Domestication /Variety). A pairwise Adonis test using a false discovery rate (FDR) p value adjustment was used for post-hoc testing at an α = 0.05.

A differential abundance (DA) analysis was run using microbiomeMarker, an R/Bioconductor package^81^, in which a Wald test was used to compare the log-fold change in bacterial abundance among domestication groups in a negative binomial distribution. An FDR p adjustment method was used to determine significant differences at an α = 0.05. Counts were normalized using the relative log expression which used the abundance mean of all samples as reference for scaling. To assess predive functions of rhizosphere bacteria in unfertilized soil, data from PICRUSt2 was explored. PICRUSt2 accurately and significantly correlates gene abundances with those values produced from gene-specific primers, such as those for the *acdS* gene^82^. However, these functions that are linked to bacteria are solely predictive^42^. To understand the different predictive functional profiles in the microbiome, a one-way ANOVA (Abundance ~ Domestication) was run on the relative abundance data of bacteria possessing targeted genes. Data for fertilized soil were partialled out. Some residual plots for bacterial abundance were not normally distributed, so for those genes (*hcnA, budA, budC, E3.2.1.6, ISC, AcP,3PH, 4PH, phlD, ituA, fenA, srfAA, rifM, phzE, mbtI, entA, pchB, E3.2.1.21, appA, phoN, nifH, nifD, nifK, nrfA, nirA, NIT-6, nirK, nirS, norB, norC*), data were log-transformed. A Tukey HSD test was used for post-hoc testing at α = 0.05, and p values were adjusted using an FDR at α = 0.05.

### Plant material statement

The plant collection and use was in accordance with all the relevant guidelines. All permissions were obtained to grow tomato and to obtain germplasm from licensed distributers. There was no formal identification of plant material used in this study by any of the authors because all plant specimens used in this study were obtained either through purchase through commercial retailers or through requisition requests from the Tomato Genetic Resource Center. Thus, there is no applicable voucher specimen to deposit into an herbarium. For more information, please see the materials and methods section.

### Supplementary Information

Supplementary Table 1.

Supplementary Table 2.

Supplementary Table 3.

Supplementary Figure 1.

Supplementary Figure 2.

Supplementary Figure 3.

Supplementary Figure 4.

## Data Availability

The data and code underlying this article are available and can be accessed at https://github.com/marydixon/Tomato-P-Recovery.
