# Supplementary material for: Tomato domestication rather than subsequent breeding events reduces microbial associations related to phosphorus recovery
Source: Sci Rep. 2024 Apr 30;14:9934. doi: 10.1038/s41598-024-60775-3 (PMC11061195; doi:10.1038/s41598-024-60775-3)
Supplement: Supplementary file 1 — Supplementary Table 1. [file 41598_2024_60775_MOESM1_ESM.pdf]

Supplemental Table 1: Domestication categorization and approximate year of development for the tested tomato accessions.

Tomato Species	Accession Name	Approximate Year Developed	Domestication Group
Solanum lycopersicum	Quali T 27	2010s	Modern
Solanum lycopersicum	Bobcat	2010s	Modern
Solanum lycopersicum	Line 1	2020s	Modern
Solanum lycopersicum	Line 2	2020s	Modern
Solanum lycopersicum	Brandywine Pink	1890s	Traditional
Solanum lycopersicum	Rutgers	1925	Traditional
Solanum lycopersicum	Marglobe	1930s	Traditional
Solanum lycopersicum	Matchless	1889	Traditional
Solanum lycopersicum var. *cerasiforme*	LA1580	Unknown	Wild
Solanum lycopersicum var. *cerasiforme*	LA1698	Unknown	Wild
Solanum pimpinellifolium	LA1519	Unknown	Wild
Solanum pennellii	LA0716	Unknown	Wild
