# Supplementary material for: Tomato domestication rather than subsequent breeding events reduces microbial associations related to phosphorus recovery
Source: Sci Rep. 2024 Apr 30;14:9934. doi: 10.1038/s41598-024-60775-3 (PMC11061195; doi:10.1038/s41598-024-60775-3)
Supplement: Supplementary file 2 — Supplementary Table 2. [file 41598_2024_60775_MOESM2_ESM.pdf]

Supplemental Table 2. Non-varied relative abundance of bacteria with predictive functions among tomatoes across a domestication gradient in unfertilized soil. Bacterial relative abundance (presented as mean ± SEM) in unfertilized soil is shown for each tomato domestication group (Modern, Traditional, Wild). An ANOVA (F and FDR-adjusted p values presented) with Tukey HSD at α = 0.05 was used to determine differences in the relative abundance of rhizosphere bacteria as a function of tomato domestication group. Different letters within each row denote significant differences in the relative abundance of the bacteria with corresponding tested gene. For data that did not have normally distributed residuals, a log-transformation was run (denoted as "L" in the F-value column). "Function" represents the predictive function for the bacteria with the corresponding gene listed in the "Gene" column. "KEGG" represents the KEGG entry used to for each corresponding gene. This table illustrates the genes are not significantly different (Table 2 shows the values for the genes that are significantly different).

Gene	Function	KEGG	F	p	Relative Abundance		
					Modern	Traditional	Wild
budA	Biocontrol	K01575	2.94^{L}	0.112	0.085 ± 0.001 a	0.085 ± 0.002 a	0.095 ± 0.004 a
budC	Biocontrol	K18009	3.62^{L}	0.072	0.008 ± 0.006 a	0.079 ± 0.002 a	0.091 ± 0.005 a
E3.2.1.14	Biocontrol	K01183	1.05	0.482	0.106 ± 0.003 a	0.166 ± 0.003 a	0.168 ± 0.005 a
ISC	Antifungal	EC.5.4.4.2_1	3.91^{L}	0.061	0.059 ± 0.001 b	0.059 ± 0.002 b	0.068 ± 0.004 a
srfAA	Antibacterial	K15654	4.34^{L}	0.057	0.058 ± 0.001 b	0.059 ± 0.002 b	0.068 ± 0.004 a
acdS	Root Growth	K01505	3.94	0.061	0.154 ± 0.002 b	0.158 ± 0.004 ab	0.170 ± 0.006 a
PCH	Chelation	EC.4.2.99.21_1	4.23	0.059	0.075 ± 0.002 b	0.074 ± 0.003 b	0.087 ± 0.005 a
E3.2.1.21	Carbon Decomposition	E3.2.1.21	3.81^{L}	0.063	0.055 ± 0.001 ab	0.055 ± 0.002 b	0.065 ± 0.004 a
ipdC	Stress	K04103	1.19	0.430	0.122 ± 0.002 a	0.121 ± 0.002 a	0.127 ± 0.004 a
entA	Siderophore	K00216	2.46^{L}	0.140	0.087 ± 0.001 a	0.092 ± 0.002 a	0.096 ± 0.004 a
nosZ	Denitrification	K00376	0.83	0.536	0.159 ± 0.002 a	0.162 ± 0.005 a	0.166 ± 0.005 a
pchB	Siderophore	K02364	2.82^{L}	0.113	0.082 ± 0.001 a	0.084 ± 0.002 a	0.092 ± 0.004 a
bglX	Carbon Decomposition	K05349	2.69	0.123	0.405 ± 0.005 a	0.417 ± 0.006 a	0.432 ± 0.012 a
bglB	Carbon Decomposition	K05350	1.44	0.356	0.358 ± 0.005 a	0.370 ± 0.005 a	0.368 ± 0.007 a
amiE	Nitrogen Decomposition	K01426	0.04	0.977	0.722 ± 0.005 a	0.723 ± 0.005 a	0.725 ± 0.007 a
phoA	Phosphorus Decomposition	K01077	0.52	0.671	0.248 ± 0.004 a	0.253 ± 0.005 a	0.255 ± 0.006 a
phoD	Phosphorus Decomposition	K01113	0.24	0.837	0.507 ± 0.004 a	0.506 ± 0.004 a	0.511 ± 0.007 a
pqqC	Phosphorus Solubilization	K06137	4.08	0.059	0.197 ± 0.003 b	0.199 ± 0.003 b	0.213 ± 0.006 a
PHO	Phosphorus Decomposition	K01078	4.14	0.059	0.107 ± 0.003 ab	0.104 ± 0.004 b	0.123 ± 0.007 a
appA	Phosphorus Decomposition	K01093	3.91^{L}	0.061	0.063 ± 0.001 b	0.064 ± 0.002 b	0.073 ± 0.004 a
pqs	P Cycling	EC.3.4.11.1_1	0.02^{L}	0.980	0.129 ± 0.002 a	0.129 ± 0.002 a	0.131 ± 0.004 a
alp	P Cycling	EC.3.1.3.1_1	0.66	0.610	0.425 ± 0.003 a	0.433 ± 0.005 a	0.425 ± 0.006 a
AcP	P Cycling	EC.3.1.3.2_1	0.43^{L}	0.711	0.071 ± 0.003 a	0.067 ± 0.002 a	0.071 ± 0.005 a
3PH	P Cycling	EC.3.1.3.8_1	0.59^{L}	0.643	0.076 ± 0.003 a	0.076 ± 0.003 a	0.083 ± 0.005 a
4PH	P Cycling	EC.3.1.3.26_1	0.42^{L}	0.711	0.010 ± 0.001 a	0.010 ± 0.000 a	0.009 ± 0.006 a
aslA	Sulfur	K01130	1.37	0.372	0.413 ± 0.006 a	0.408 ± 0.005 a	0.398 ± 0.008 a
