# Supplementary material for: Tomato domestication rather than subsequent breeding events reduces microbial associations related to phosphorus recovery
Source: Sci Rep. 2024 Apr 30;14:9934. doi: 10.1038/s41598-024-60775-3 (PMC11061195; doi:10.1038/s41598-024-60775-3)
Supplement: Supplementary file 3 — Supplementary Table 3. [file 41598_2024_60775_MOESM3_ESM.pdf]

Supplemental Table 2. KEGG orthologs used for predictive function identification. References used to support the function mentioned in the main text are provided in the "Reference" column.

Enzyme	Gene	Function	KEGG	Reference
Acetolactate decarboxylase	*budA*	Biocontrol	K01575	Chlebek, D., *et al.* Genetic Determinants of Antagonistic Interactions and the Response of New Endophytic Strain Serratia quinivorans KP32 to Fungal Phytopathogens. *International Journal of Molecular Sciences* 2022;23(24):15561.
(S,S)-butanediol dehydrogenase	*budC*	Biocontrol	K18009	Dudeja, S.S., *et al.* Bacterial endophytes: molecular interactions with their hosts. *Journal of Basic Microbiology* 2021;61(6):475-505.
Chitinase	*E3.2.1 .14*	Biocontrol	K01183	Gomaa, E.Z. Chitinase production by Bacillus thuringiensis and Bacillus licheniformis: their potential in antifungal biocontrol. *The journal of Microbiology* 2012;50:103-111.
Isochorismate synthase	*ISC*	Antifungal	EC.5.4.4.2_ 1	Perez, E., *et al.* The importance of chorismate mutase in the biocontrol potential of Trichoderma parareesei. *Frontiers in Microbiology* 2015;6:1181.
Surfactin family lipopeptide synthetase A	*srfAA*	Antibacterial	K15654	Mora, I., Cabrefiga, J. and Montesinos, E. Cyclic lipopeptide biosynthetic genes and products, and inhibitory activity of plant-associated Bacillus against phytopathogenic bacteria. *PLoS One* 2015;10(5):e0127738.
1-aminocyclopropane-1-carboxylate deaminase	*acdS*	Root Growth	K01505	Manter, D.K., Hamm, A.K. and Deel, H.L. Community structure and abundance of ACC deaminase containing bacteria in soils with 16S-PICRUSt2 inference or direct acdS gene sequencing. *Journal of Microbiological Methods* 2023:106740.
Isochorismate lyase	*PCH*	Chelation	EC.4.2.99.2 1_1	Cunrath, O., *et al.* A cell biological view of the siderophore pyochelin iron uptake pathway in p seudomonas aeruginosa. *Environmental microbiology* 2015;17(1):171-185.
Beta-glucosidase	*E3.2.1 .21*	Carbon Decomposition	E3.2.1.21	Adetunji, A.T., *et al.* The biological activities of β-glucosidase, phosphatase and urease as soil quality indicators: a review. *Journal of soil science and plant nutrition* 2017;17(3):794-807.
Indolepyruvate decarboxylase	*ipdC*	Stress	K04103	Malhotra, M. and Srivastava, S. Organization of the ipdC region regulates IAA levels in different Azospirillum brasilense strains: molecular and functional analysis of ipdC in strain SM. *Environmental Microbiology* 2008;10(5):1365-1373.
2,3-dihydro-2,3-dihydroxybenzoate dehydrogenase	*entA*	Siderophore	K00216	Penwell, W.F., Arivett, B.A. and Actis, L.A. The Acinetobacter baumannii entA gene located outside the acinetobactin cluster is critical for siderophore production, iron acquisition and virulence. *PloS one* 2012;7(5):e36493.
Nitrous-oxide reductase	*nosZ*	Denitrification	K00376	Dandie, C., *et al.* Analysis of denitrification genes and comparison of nosZ, cnorB and 16S rDNA from culturable denitrifying bacteria in potato cropping systems. *Systematic and applied microbiology* 2007;30(2):128-138.
Isochorismate pyruvate lysase	*pchB*	Siderophore	K02364	Olucha, J.: University of Kansas; 2012. Structural, Functional and Computational Characterization of Pseudomonas aeruginosa Siderophore Biosynthetic Pathway Accessory Proteins PchB and PvdA.
Beta-glucosidase	*bglX*	Carbon Decomposition	K05349	Adetunji, A.T., *et al.* The biological activities of β-glucosidase, phosphatase and urease as soil quality indicators: a review. *Journal of soil science and plant nutrition* 2017;17(3):794-807.
Beta-glucosidase	*bglB*	Carbon Decomposition	K05350	Adetunji, A.T., *et al.* The biological activities of β-glucosidase, phosphatase and urease as soil quality indicators: a review. *Journal of soil science and plant nutrition* 2017;17(3):794-807.
Amidase	*amiE*	Nitrogen Decomposition	K01426	Ochiai, S., *et al.* AmiE, a novel N-acylhomoserine lactone acylase belonging to the amidase family, from the activated-sludge isolate Acinetobacter sp. strain Ooi24. *Applied and Environmental Microbiology* 2014;80(22):6919-6925.
Alkaline phosphatase	*phoA*	Phosphorus Decomposition	K01077	Orchard, E., Webb, E. and Dyhrman, S. Characterization of phosphorus-regulated genes in Trichodesmium spp. *The Biological Bulletin* 2003;205(2):230-231.
Alkaline	*phoD*	Phosphorus	K01113	Zhu, X., *et al.* Distribution characteristics of phoD-harbouring

phosphatase		Decomposition		bacterial community structure and its roles in phosphorus transformation in steppe soils in Northern China. *Journal of Soil Science and Plant Nutrition* 2021;21:1531-1541.
Pyrroloquinoline-quinone synthase	*pqqC*	Phosphorus Solubilization	K06137	Misra, H., Rajpurohit, Y. and Khairnar, N. Pyrroloquinoline-quinone and its versatile roles in biological processes. *Journal of biosciences* 2012;37:313-325.
Acid phosphatase	*PHO*	Phosphorus Decomposition	K01078	Hammond, J.P., Broadley, M.R. and White, P.J. Genetic responses to phosphorus deficiency. *Annals of botany* 2004;94(3):323-332.
Acid phosphatase	*appA*	Phosphorus Decomposition	K01093	Touati, E. and Danchin, A. Cloning and characterization of the pH 2.5 acid phosphatase gene, appA: cyclic AMP mediated negative regulation. *Molecular and General Genetics MGG* 1987;208:499-505.
Pyrroloquinoline-quinone synthase	*pqs*	P Cycling	EC.3.4.11.1_1	Misra, H., Rajpurohit, Y. and Khairnar, N. Pyrroloquinoline-quinone and its versatile roles in biological processes. *Journal of biosciences* 2012;37:313-325.
Alkaline phosphatase	*alp*	P Cycling	EC.3.1.3.1_1	Sharma, U., Pal, D. and Prasad, R. Alkaline phosphatase: an overview. *Indian journal of clinical biochemistry* 2014;29:269-278.
Acid phosphatase	*AcP*	P Cycling	EC.3.1.3.2_1	Fuchs, K.R., Shekels, L.L. and Bernlohr, D.A. Analysis of the ACP1 gene product: classification as an FMN phosphatase. *Biochemical and biophysical research communications* 1992;189(3):1598-1605.
3-phytase	*3PH*	P Cycling	EC.3.1.3.8_1	Sajidan, A., *et al.* Molecular and physiological characterisation of a 3-phytase from soil bacterium Klebsiella sp. ASR1. *Applied microbiology and biotechnology* 2004;65:110-118.
4-phytase	*4PH*	P Cycling	EC.3.1.3.26_1	Ranjan, K. and Sahay, S. Identification of phytase producing yeast and optimization and characterization of extracellular phytase from Candida parapsilosis. *Int J Sci Nat* 2013;4(4):583-590.
Arylsulfatase	*aslA*	Sulfur Decomposition	K01130	Cregut, M. and Rondags, E. New insights in agar biorefinery with arylsulphatase activities. *Process Biochemistry* 2013;48(12):1861-1871.
Methane/ammonia monooxygenase	*pmoA-amoA*	Nitrification	K10944	Van Kessel, M.A., *et al.* Complete nitrification by a single microorganism. *Nature* 2015;528(7583):555-559.
Nitrogenase Fe protein	*nifH*	Nitrogen Fixation	K02588	Gaby, J.C. and Buckley, D.H. A comprehensive evaluation of PCR primers to amplify the nifH gene of nitrogenase. 2012.
Nitrogenase Mo-Fe protein	*nifK*	Nitrogen Fixation	K02586	Fani, R., Gallo, R. and Liò, P. Molecular evolution of nitrogen fixation: the evolutionary history of the nifD, nifK, nifE, and nifN genes. *Journal of molecular evolution* 2000;51:1-11.
Nitrogenase Mo-Fe protein	*nifD*	Nitrogen Fixation	K02591	Fani, R., Gallo, R. and Liò, P. Molecular evolution of nitrogen fixation: the evolutionary history of the nifD, nifK, nifE, and nifN genes. *Journal of molecular evolution* 2000;51:1-11.
Hydroxylamine dehydrogenase	*hao*	Nitrification	K10535	Bergmann, D.J., Hooper, A.B. and Klotz, M.G. Structure and sequence conservation of hao cluster genes of autotrophic ammonia-oxidizing bacteria: evidence for their evolutionary history. *Applied and environmental microbiology* 2005;71(9):5371-5382.
Nitrite reductase (NADH)	*nirD*	Dissimilatory Nitrate Reduction	K00363	Hu, R., *et al.* Evidence for assimilatory nitrate reduction as a previously overlooked pathway of reactive nitrogen transformation in estuarine suspended particulate matter. *Environmental Science & Technology* 2022;56(20):14852-14866.
Nitrite reductase (cytochrome c-552)	*nrfA*	Dissimilatory Nitrate Reduction	K03385	Hu, R., *et al.* Evidence for assimilatory nitrate reduction as a previously overlooked pathway of reactive nitrogen transformation in estuarine suspended particulate matter. *Environmental Science & Technology* 2022;56(20):14852-14866.
Ferrodoxin-nitrite reductase	*nirA*	Assimilatory Nitrate Reduction	K00366	Frías, J.E. and Flores, E. Induction of the nitrate assimilation nirA operon and protein-protein interactions in the maturation of nitrate and nitrite reductases in the cyanobacterium Anabaena sp. strain PCC 7120. *Journal of Bacteriology* 2015;197(14):2442-2452.
Nitrate reductase	*narG*	Denitrification	K00370	Chèneby, D., *et al.* Genetic characterization of the nitrate reducing community based on narG nucleotide sequence analysis. *Microbial ecology* 2003;46(1):113-121.

Nitrate reductase	*narH*	Denitrification	K00371	Petri, R. and Imhoff, J.F. The relationship of nitrate reducing bacteria on the basis of narH gene sequences and comparison of narH and 16S rDNA based phylogeny. *Systematic and Applied Microbiology* 2000;23(1):47-57.
Nitrite reductase (NO forming)	*nirK*	Denitrification	K00368	Dandie, C., *et al.* Analysis of denitrification genes and comparison of nosZ, cnorB and 16S rDNA from culturable denitrifying bacteria in potato cropping systems. *Systematic and applied microbiology* 2007;30(2):128-138.
Nitric oxide reductase	*norB*	Denitrification	K04561	Schmidt, I., van Spanning, R.J. and Jetten, M.S. Denitrification and ammonia oxidation by Nitrosomonas europaea wild-type, and NirK-and NorB-deficient mutants. *Microbiology* 2004;150(12):4107-4114.
Glycine dehydrogenase	*hcnA*	Chelation	K10814	Rijavec, T. and Lapanje, A. Hydrogen cyanide in the rhizosphere: not suppressing plant pathogens, but rather regulating availability of phosphate. *Frontiers in microbiology* 2016;7:1785.
Phloroglucinol synthase	*phlD*	Antifungal	K15431	Yang, F. and Cao, Y. Biosynthesis of phloroglucinol compounds in microorganisms. *Applied Microbiology and Biotechnology* 2012;93:487-495.
Iturin family lipopeptide synthetase A	*ituA*	Antifungal	K15661	Dang, Y., *et al.* Enhanced production of antifungal lipopeptide iturin A by Bacillus amyloliquefaciens LL3 through metabolic engineering and culture conditions optimization. *Microbial cell factories* 2019;18:1-14.
Fengycin family lipopeptide synthetase D	*fenA*	Antifungal	K15667	Moyne, A.-L., Cleveland, T.E. and Tuzun, S. Molecular characterization and analysis of the operon encoding the antifungal lipopeptide bacillomycin D. *FEMS Microbiology Letters* 2004;234(1):43-49.
AHBA synthesis associated protein	*rifM*	Antibiotic	K16017	Chen, S., *et al.* Biosynthesis of ansatrienin (mycotrienin) and naphthomycin: identification and analysis of two separate biosynthetic gene clusters in Streptomyces collinus Tü 1892. *European journal of biochemistry* 1999;261(1):98-107.
2-amino-4-deoxychorismate synthase	*phzE*	Antibiotic	K13063	McDonald, M., *et al.* Phenazine biosynthesis in Pseudomonas f luorescens: Branchpoint from the primary shikimate biosynthetic pathway and role of phenazine-1, 6-dicarboxylic Acid. *Journal of the American Chemical Society* 2001;123(38):9459-9460.
Salicylate synthetase	*mbtI*	Chelation	K04781	Harrison, A.J., *et al.* The structure of MbtI from Mycobacterium tuberculosis, the first enzyme in the biosynthesis of the siderophore mycobactin, reveals it to be a salicylate synthase. *Journal of bacteriology* 2006;188(17):6081-6091.
Isochorismatase	*entB*	Chelation	EC.3.3.2.1_1	Chlebek, D., *et al.* Genetic Determinants of Antagonistic Interactions and the Response of New Endophytic Strain Serratia quinivorans KP32 to Fungal Phytopathogens. *International Journal of Molecular Sciences* 2022;23(24):15561.
Urease alpha subunit	*ureC*	Nitrogen Decomposition	K01428	Adetunji, A.T., *et al.* The biological activities of β-glucosidase, phosphatase and urease as soil quality indicators: a review. *Journal of soil science and plant nutrition* 2017;17(3):794-807.
Acid phosphatase	*phoN*	Phosphorus Decomposition	K09474	Adetunji, A.T., *et al.* The biological activities of β-glucosidase, phosphatase and urease as soil quality indicators: a review. *Journal of soil science and plant nutrition* 2017;17(3):794-807.
Nitrite reductase (NAD(P)H)	*NIT-6*	Assimilatory Nitrate Reduction	K00366	Besson, S., Almeida, M.G. and Silveira, C.M. Nitrite reduction in bacteria: A comprehensive view of nitrite reductases. *Coordination Chemistry Reviews* 2022;464:214560.
Nitrite reductase (NO forming)	*nirS*	Denitrification	K15864	Braker, G. and Tiedje, J.M. Nitric oxide reductase (norB) genes from pure cultures and environmental samples. *Applied and environmental microbiology* 2003;69(6):3476-3483.
Nitric oxide reductase	*norC*	Denitrification	K02305	Braker, G. and Tiedje, J.M. Nitric oxide reductase (norB) genes from pure cultures and environmental samples. *Applied and environmental microbiology* 2003;69(6):3476-3483.
