# Supplementary material for: Tomato domestication rather than subsequent breeding events reduces microbial associations related to phosphorus recovery
Source: Sci Rep. 2024 Apr 30;14:9934. doi: 10.1038/s41598-024-60775-3 (PMC11061195; doi:10.1038/s41598-024-60775-3)
Supplement: Supplementary file 4 — Supplementary Figure 1. [file 41598_2024_60775_MOESM4_ESM.pdf]

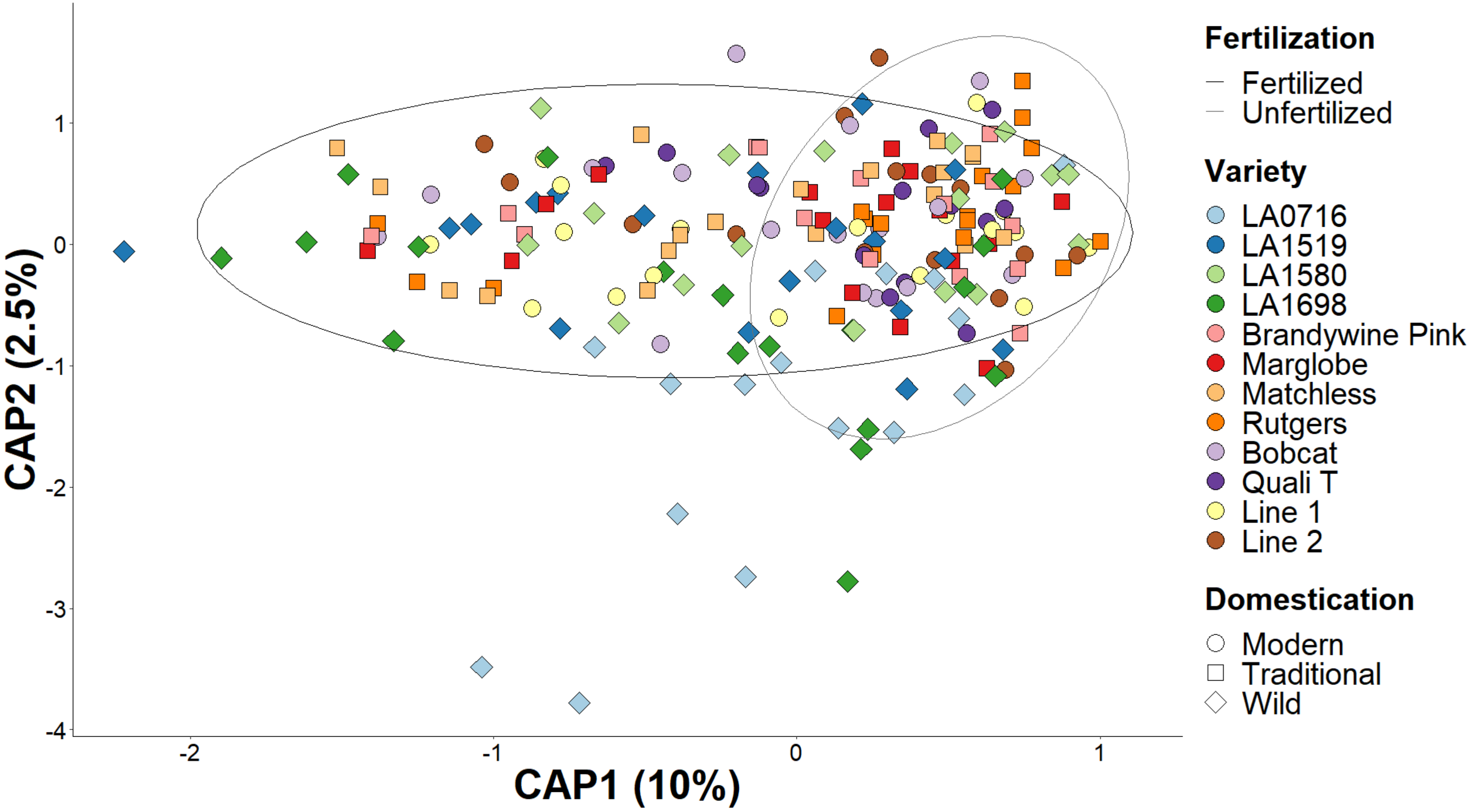

Supplemental Figure 1. Distance-based redundancy analysis (db-RDA) showing the clustering based on Bray-Curtis dissimilarity of the bacterial community structure in the tomato rhizosphere. The ellipses represent the fertilization treatment: fertilized (black) and unfertilized (gray). The color represents the variety: LA0716 (light blue), LA1519 (dark blue), LA1580 (light green), LA1698 (dark green), 'Brandywine Pink' (pink), 'Marglobe' (red), 'Matchless' (light orange), 'Rutgers' (dark orange), 'Bobcat' (light purple), 'Quali T' (dark purple), Line 1 (yellow), Line 2 (brown). The shape represents the level of domestication: modern (circle), traditional (square), and wild (diamond).
