# Supplementary material for: Tomato domestication rather than subsequent breeding events reduces microbial associations related to phosphorus recovery
Source: Sci Rep. 2024 Apr 30;14:9934. doi: 10.1038/s41598-024-60775-3 (PMC11061195; doi:10.1038/s41598-024-60775-3)
Supplement: Supplementary file 5 — Supplementary Figure 2. [file 41598_2024_60775_MOESM5_ESM.pdf]

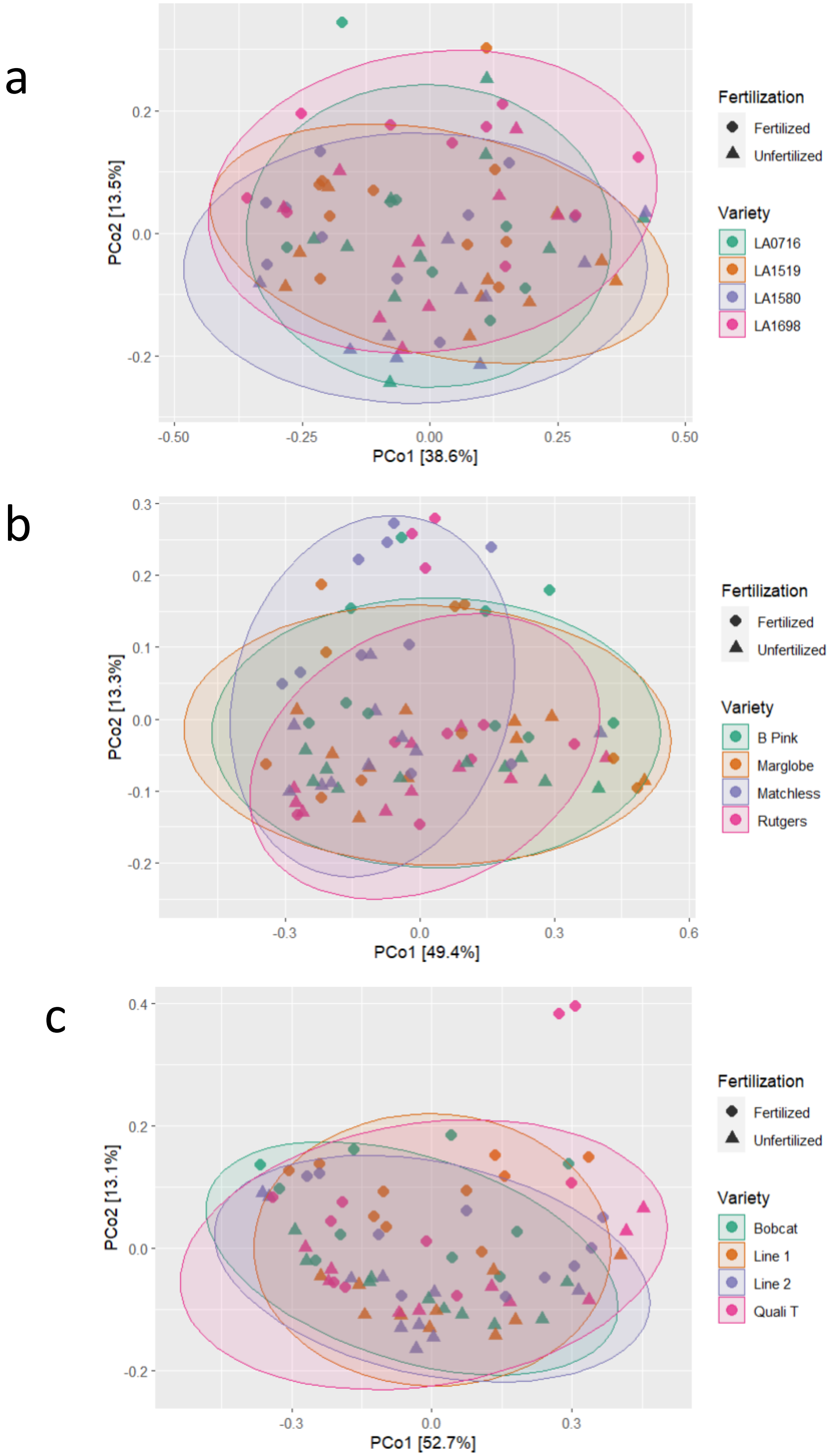

Supplemental Figure 2.Principal coordinate analysis (PCoA) clustering of the bacterial community structure in the tomato rhizosphere. The ellipses and colors indicate the different tomato varieties: LA0716, 'Brandywine Pink', 'Bobcat' (yellow); LA1519, 'Marglobe', 'Quali T' (light blue); LA1580, 'Matchless', Line 1 (dark blue); LA1698, 'Rutgers', V8053 (red). The shapes represent the fertilization treatment: fertilized (circle) and unfertilized (triangle).
