# Supplementary material for: Tomato domestication rather than subsequent breeding events reduces microbial associations related to phosphorus recovery
Source: Sci Rep. 2024 Apr 30;14:9934. doi: 10.1038/s41598-024-60775-3 (PMC11061195; doi:10.1038/s41598-024-60775-3)
Supplement: Supplementary file 6 — Supplementary Figure 3. [file 41598_2024_60775_MOESM6_ESM.pdf]

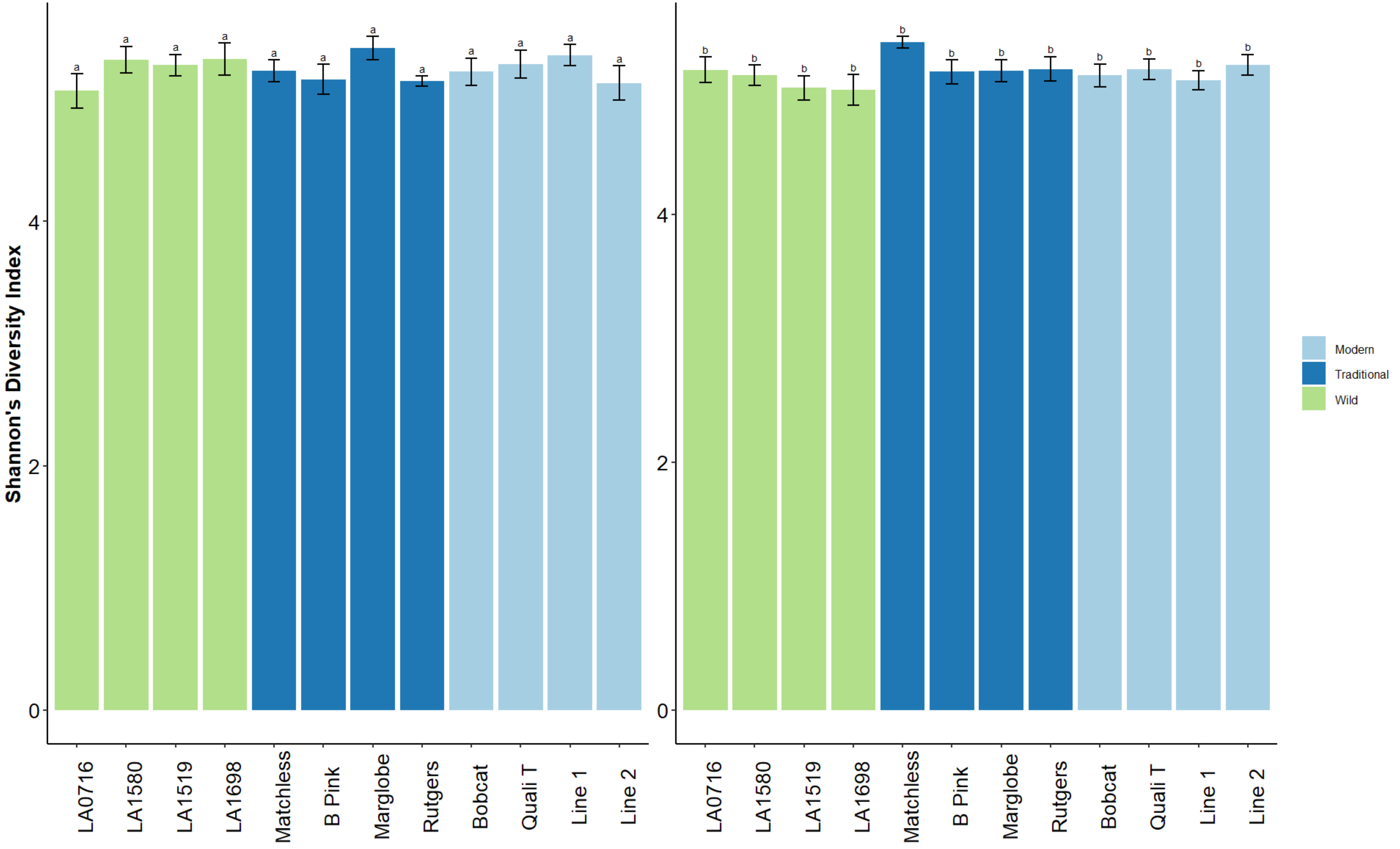

Supplemental Figure 3. Shannon's diversity index of tomatoes across a domestication gradient. Different colored bars indicate the domestication level: wild (green), traditional (dark blue), and modern (light blue). An ANOVA was run with a Tukey HSD test for post-hoc comparison. Different lowercase letters denote significant differences ($p < 0.05$) for the fertilization level: left panel indicates fertilized tomatoes and right panel indicates unfertilized tomatoes. There were no significant differences among tomato variety or domestication group ($p > 0.05$).
