# Supplementary material for: Tomato domestication rather than subsequent breeding events reduces microbial associations related to phosphorus recovery
Source: Sci Rep. 2024 Apr 30;14:9934. doi: 10.1038/s41598-024-60775-3 (PMC11061195; doi:10.1038/s41598-024-60775-3)
Supplement: Supplementary file 7 — Supplementary Figure 4. [file 41598_2024_60775_MOESM7_ESM.pdf]

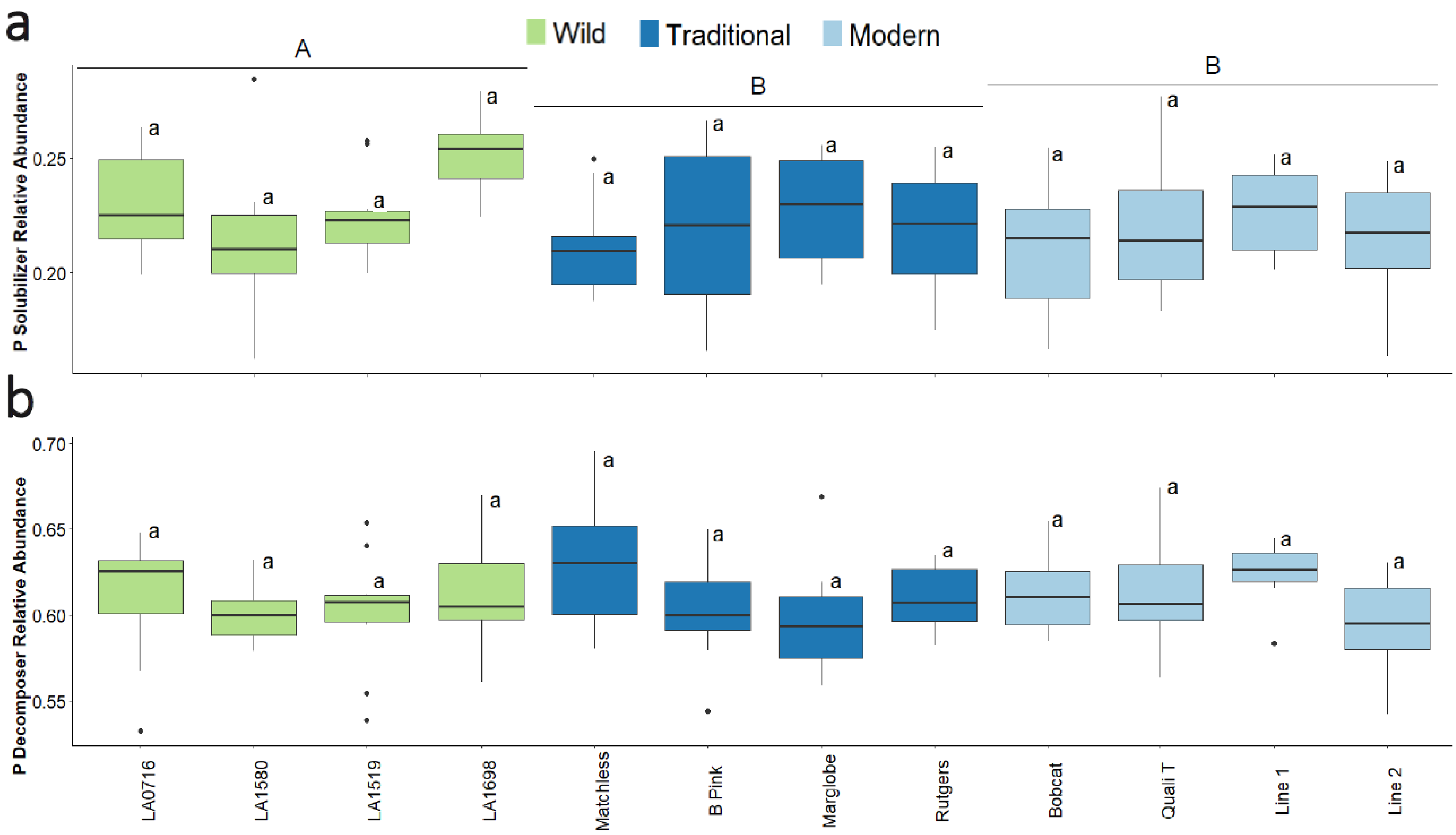

Supplemental Figure 3. Phosphorus (P) solubilizing and P decomposing bacteria relative abundance in fertilized soil.
a *P solubilizing bacteria in fertilized soil. An ANVOA showed differences in shoot P uptake for Domestication main effect.*
b *P decomposing bacteria in unfertilized soil. An ANVOA showed no differences in shoot P uptake for any tested effect.*
Different colored bars indicate the domestication level: wild (green), traditional (dark blue), and modern (light blue). An ANOVA was run with a Tukey HSD test for post-hoc comparison. Different lowercase letters denote significant differences ($p < 0.05$) for the different varieties. Different uppercase letters denote significant differences ($p < 0.05$) for the different domestication groups. Treatments sharing a common letter are not significantly different.
